# Selecting suitable entities to implement blockchain in green agricultural supply chains

**DOI:** 10.1371/journal.pone.0334867

**Published:** 2026-07-06

**Authors:** Guangxing Wei, Jiayi Shen, Xiaohan Guo

**Affiliations:** School of Economics and Management, Chongqing Jiaotong University, Chongqing, China; Sapienza University of Rome: Universita degli Studi di Roma La Sapienza, ITALY

## Abstract

Blockchain implementation in green agricultural supply chains can be undertaken independently by manufacturers or retailers or jointly by both in industrial practices. However, most existing studies predesignate implementation entities artificially and largely ignore firms’ endogenous economic willingness, which restricts the practical explanatory capability. Addressing this research gap, this study systematically explores firms’ implementation willingness and the optimal selection of blockchain implementers in green agricultural supply chains, thereby offering targeted decision-making insights for blockchain policymakers and industrial practitioner. Four game-theoretic models are constructed involving entities: none, retailer-only, manufacturer-only, and both. By comparing the equilibrium outcomes of different game models, this study employs the dual thresholds of deployment costs and operational costs to quantitatively evaluate the economic willingness of supply chain entities to implement blockchain. Different from prior studies that treat blockchain implementers as exogenous and only consider a single cost dimension, this paper endogenizes firms’ blockchain implementation decisions based on economic intentions and establishes a dual-cost threshold framework to categorize and identify the optimal implementer under different scenarios, and illustrate the results with a case study. The key findings are concluded as follows. First, economic willingness varies within thresholds. Manufacturers and retailers may take the initiative to implement blockchain regardless of their partners’ behavior, or act reactively by following their partners; beyond the thresholds, they refuse to implement it. Second, entity selection varies across scenarios. Under moderately high dual costs, independent retailer implementation is optimal; under balanced costs, manufacturers may implement alone. Extremely high operational costs lead to no willing implementer, while extremely high deployment costs require joint implementation. Third, the likelihood of implementation modes differs substantially within thresholds. The most likely scenario is retailers’ independent implementation, the least likely is manufacturers’ independent implementation, with joint implementation in between. Beyond these thresholds, no entity implements it at all, a situation accounting for over 50% in the case study. Methodologically, this paper applies global optimization rather than the local optimization strategy with fixed designated implementer settings in previous studies, effectively capturing the internal economic motivation driving voluntary blockchain implementation in green agricultural supply chains.

## 1. Introduction

Agricultural supply chains are undergoing profound green transformation required by the United Nations 2030 Agenda for Sustainable Development. However, the long-distance and time-consuming circulation of agricultural products triggers information distrust regarding product greenness because every supply chain link may affect the credibility of green products [1]. Adopting blockchain technology is essential to improve transparency and traceability within green agricultural supply chains [2]. For example, HUAWEI empowers the whole industrial chain of rice supply chains by embedding blockchain into the entire process of production, processing, circulation, and marketing, where core data covering land use, seed breeding, pesticide and fertilizer application, harvesting, and logistics are uploaded to a blockchain system to guarantee product quality and origin traceability.

In practice, green agricultural supply chains implement blockchain in three typical modes. First, upstream manufacturers implement blockchain independently. As a typical agricultural enterprise, China Green Foods Group uploads full lifecycle farm-to-table data onto the blockchain to realize real-time quality supervision of agricultural products. Second, downstream retailers implement blockchain alone. As a leading domestic supermarket brand, Yong-Hui Superstores employs blockchain to archive production and quarantine information, assigning a unique traceability code to each turbot product. Third, upstream manufacturers and downstream retailers jointly deploy blockchain. For example, Wuchang Farmer Cooperative collaborates with Tmall and Cai-Niao Logistics as the retailers to apply blockchain traceability to the Wuchang rice supply chain, where each rice bag in the official Tmall flagship store is labeled with an exclusive Quick Response code for full lifecycle traceability.

Although practical blockchain adoption covers three typical modes, extant literature has hardly examined the essential question of why blockchain technology is implemented by manufacturers or retailers individually in some situations and jointly by both parties in others. That is, who should be selected as the appropriate blockchain implementer and under what conditions are manufacturers and retailers willing to implement blockchain in green agricultural supply chains? The entities implementing blockchain in supply chains are often mandatorily designated without considering their economic willingness, although they are only willing to implement voluntarily when they expect higher economic profits. For example, the upstream manufacturer is subjectively assumed to implement blockchain solely in [3], while in [4] it is assumed to do so cooperatively with the downstream retailer and platform, without any explanation for the different settings. With these predefined entities, previous studies have deeply examined how blockchain enhances operational efficiency [5] and information transparency [6] in supply chains, while ignoring firms’ economic willingness and voluntary adoption motivation. But preconditional questions such as whether the designated entities are willing to adopt blockchain, have hardly been probed. There is a significant gap between industrial practice and theoretical research, which is practically meaningful and academically valuable. To bridge this gap, this paper sequentially explores the questions as follows.

1) What differences exist in the impacts of blockchain adoption when it is implemented individually by manufacturers, individually by retailers, or jointly by both parties within green agricultural supply chains?
2) What is the economic willingness of each entity to implement blockchain technology in green agricultural supply chains, whether initiative or reactive?
3) Which entity or entities should be contextually selected to implement blockchain technology in green agricultural supply chains under various conditions?

To address the above issues, this paper constructs four game-theoretic models featuring different entities: none, the retailer alone, the manufacturer only, and both of them. By solving the models and comparing profits across them, the economic willingness of each entity is quantified through the dual-cost thresholds incorporating the fixed deployment costs and variable operational costs of blockchain implementation. The optimal entity selection is explored and illustrated with a visually case study in each scenario, which are classified by the criteria of dual-cost thresholds.

The coming rest is structured as follows. Section 2 reviews previous literature and specifies the research gap. Section 3 presents the problem description and necessary assumptions, and Section 4 develops and solves game theoretic models. Section 5 probes the economic willingness to implement blockchain, and Section 6 examines the entity selection in each scenario, while Section 7 presents visualized verification and Section 8 draws conclusions.

## 2. Literature review

This paper explores the issues of economic willingness and entity selection of implementing blockchain in green agricultural supply chains. Following the common methodological guidance in literature such as [7,8], we categorize the related literature into three streams: the roles, entities, and costs of implementing blockchain in supply chains across general supply chains including agriculture. To begin with, the research landscape and limitations are summarized in the following Table 1.

**Table 1:** Outline of previous literature on implementing blockchain in supply chains.

Research streams	Sub-streams	Research limitations
Roles	Promoting supply chain performance.	Few studies probed these two aspects simultaneously.
	Enhancing information transparency.	
Entities	Single entity: e.g., either manufacturer or retailer implements blockchain independently.	Previous literature has never explored whether given entities are willing to adopt blockchain voluntarily.
	Multiple entities: e.g., both manufacturer and retailer implement blockchain jointly.	
Costs	Single cost: examining only one side of the fixed and variable costs.	Little literature has examined both fixed and variable costs, and fails to identify their dual interaction thresholds.
	Dual costs: examining both the fixed costs and variable costs.	

### 2.1. Roles of implementing blockchain in supply chains

The related literature about the roles of implementing blockchain technology in various supply chains can be divided into two sub-streams: 1) promoting supply chain performance; 2) enhancing information transparency.

In the first sub-strand of promoting performance, empirical evidences and analytical supports have consistently found that blockchain implementation improves supply chain performance significantly. Firstly, some studies offer empirical evidence. A survey of 328 valid responses from senior managers in Vietnamese enterprises shown that blockchain implementation significantly enhances the overall efficiency of green supply chains [9]. 387 valid replies from mid-sized and large logistics corporations based in four primary national logistics hub cities of China demonstrated that blockchain implementation enhances the systematic resilience and sustainability of regional supply chains [10]. The data from listed manufacturing corporations and their partners along supply chains shown that the deployment of a blockchain system can improve the financial stability within supply chain resilience [11]. The empirical data from researchers of the United Nations proved that adopting blockchain optimizes the upstream supplier and operational performance in agri-food supply chains [12]. Taking Tesla’s battery collection and remanufacturing operations in China as a case study, [13] revealed that blockchain adoption integrated with the effect of scale economy reduced costs by over 81%. Secondly, other studies provide analytical support. In platform supply chains, implementing blockchain enhanced product greenness, the profits of the manufacturer, and those of the platform [14]. Especially, a blockchain platform improved the whole supply chain surplus and promote greater sustainability and green investment in agricultural supply chains [15]. Even if only the retailer implemented blockchain independently, the whole supply chain performance can be improved, which was determined by various factors, for instance, the substitutability of products [16]. Through evolutionary game theory, [17] illustrated that AI-blockchain synergy reduced agency costs of resilient agricultural supply chains. Generally, the functions of implementing blockchain in various supply chains were similar, such as enhancing operational efficiency and promoting collaboration in construction supply chains [18], and improving both the profitability and sustainability of maritime supply chains [19].

In the second sub-stream of enhancing transparency, empirical studies have proven that blockchain implementation improves information transparency. Survey data from 275 industry experts found that blockchain implementation can enhance transparency and security while driving sustainability in food supply chains [6]. In power battery supply chains [20], blockchain implementation enhanced traceability and discloses the information about recycled power batteries, which may either be fully reused or completely dismantled for raw materials. The implementation of blockchain incentivized real-time transparent data in low-carbon supply chains, by which consumers can access product information promptly and precisely [21]. Similarly, blockchain implementation eliminated consumers’ inaccurate judgements about product quality in e-commerce supply chains [22], increased the information disclosure level in both dual-channel supply chains [23], and promoted the elimination of information asymmetry within supply chains [24]. Especially, in agrifood supply chains [25], traceability and transparency have been proven to be the most important enablers of blockchain technology. The empirical survey of 67 Italian wine agribusinesses and a deep interview on practical blockchain industry projects, shown that the information transparency and trustworthiness in the Italian wine supply chain have been improved by blockchain systems [26].

Clearly, the extant literature has focused on performance improvement and transparency enhancement through blockchain implementation in various supply chains. However, few studies have probed these two aspects simultaneously, and even fewer have specifically targeted agricultural supply chains. Aiming at this gap, this paper examines the above two aspects from the perspective of blockchain implementation in the context of green agricultural supply chains.

### 2.2. Entities of implementing blockchain in supply chains

The related literature about the entities of implementing blockchain in supply chains may be cataloged into two strands: 1) single entity, and 2) multiple entities.

In the first sub-strand of the single entity, although blockchain technology is implemented by either the upstream entity such as a manufacturer or the downstream entity such as a retailer, and the related costs could be shared by them. For example, in semiconductor chip supply chains [3], it was the manufacturer who solely implemented blockchain, and the retailer may share some implementation costs. In remanufacturing supply chains [27], it was the original equipment manufacturer who unilaterally implemented blockchain, but the remanufactured parts supplier did not share any implementation costs. In e-commerce platform supply chains [28], the platform, as the implementer of blockchain, leveraged blockchain to promote or to abandon its adoption to prevent manufacturers’ encroachment. In the closed-loop supply chain of power batteries [20], either the manufacturer implemented blockchain independently or the manufacturer collaborated with the retailer. In the capital-constrained recycler in closed-loop supply chain, the threshold for the recycler to implement blockchain became higher under the manufacturer-dominated recycling, which offered greater potential for blockchain applications [29].

In the second more popular sub-strand of the multiple entities, blockchain technology is implemented by both the upstream entity such as a manufacturer and the downstream entity such as a retailer, and the related costs are shared by them. For instance, in dual-channel supply chains [30], manufacturer deployed blockchain covering both sales channels, while retailers limited its usage solely to the retail channel under matching conditions. In low-carbon e-commerce supply chains with oligopoly markets [31], both manufacturers implemented blockchain simultaneously when their competition was low; moreover, only under an extremely low degree of competition can all members reach a stable strategy. Blockchain technology can be implemented by either the government, the manufacturer or the retailer in low-carbon supply chains, where the retailer’s implementation surpassed other modes because it delivered the most significant sustainability improvement and achieved the highest social welfare for the entire supply chain [32]. Furthermore, it was possible that either manufacturers or retailers were willing to implement blockchain within their respective thresholds, and these entities may fall into an equilibrium of prisoners’ dilemma, that is, both wanted to free-ride on the implementation of blockchain, but finally neither implemented it at all [33]. In dual-channel supply chains, [23] examined three types of entities implementing blockchain: the upstream supplier’s independent implementation by deciding its information nodes, the downstream retailer’s independent implementation, and their simultaneous implementation where they decided their own information nodes. It was found that the incentive for the upstream supplier to implement blockchain was strong while that for the retailer relied on various factors such as competitive strength.

The above literature has consistently assumed that supply chain entities implementing blockchain—whether single or multiple—are given, which implies that these entities do not actually act voluntarily but are compelled to accept mandatory implementation, ignoring their intrinsic willingness. However, the question whether given entities are willing to adopt blockchain out of voluntary behavior has never explored. Importantly, only when some entities willingly implement it in supply chains can the blockchain system enhance supply chain performance and improve information transparency. Conversely, if any entity resists, the basis and preconditions for blockchain implementation collapse, and thus it is irrational to discuss the roles of implementing blockchain anymore. Aiming at such a potential contradiction, this paper explores the intrinsic intention of whether the manufacturer or retailer is willing to implement blockchain independently, or both are willing to act cooperatively in green agricultural supply chains.

### 2.3. Costs of implementing blockchain in supply chains

The costs of implementing blockchain technology are usually divided into fixed deployment costs and variable operational costs [34], which do not change or change with the quantity of products, respectively. On the base of cost classification, we classify the research about the costs of implementing blockchain in supply chains into two sub-streams: 1) single-type cost analysis, and 2) dual-type cost analysis.

In the first sub-strand of single-type cost analysis, either the fixed or variable costs is explored. Firstly, some studies focus solely on fixed costs. For example, in platform supply chains [35], the implementation costs borne by the manufacturer were specified as a convex function of the level of traceability, which may be shared by the retailer through a cost-sharing mechanism. In dual-channel supply chains, the implementation costs were specified as a convex function of the number of information nodes linked to the blockchain system [23]. Secondly, other studies focus solely on variable costs. For example, in multichannel supply chains [36], the supplier had to bear unit operational costs when implementing blockchain technology. Similarly, in green supply chains, [37] defined a variable cost as the unit blockchain adoption costs, which referred to the payment the manufacturer must make for each piece of product that is registered, displayed and sold via the blockchain system.

In the second sub-strand of dual-type cost analysis, limited studies have examined both the fixed costs and variable costs of implementing blockchain simultaneously. For instance, [33] classified the costs of blockchain implementation into investment costs and unit operating costs. Investment costs were specified as a convex function of the number of nodes collecting data, which determined the scale and thus shaped the scope of the blockchain system, while unit operating costs referred to the cost of labelling a code to each piece of product. In maritime supply chains, [38] probed how the blockchain’s fixed expenses such as procuring corresponding equipment, and the blockchain’s unit costs such as uploading, storing, and maintaining data affected the conditions of blockchain adoption. It was found that not only the involved costs but also the future demand can influence the compatibility of blockchain. [32] conducted an in-depth analysis of the costs of implementing blockchain in low-carbon supply chains, which were categorized into three types. The setup costs were specified as a convex function of efficiency improvement in direct emission reduction. The operational costs reflected the resource consumption required for uploading, storing, and processing related data. The usage fees represented the payments for recording emission reduction activities. The latter two actually fell under the variable cost category.

Previous literature has mainly explored only a single dimension of the fixed and variable blockchain implementation costs. Although few have examined both fixed and variable costs simultaneously, these studies still fail to identify the interactive threshold combining the dual dimension. Aiming at this gap, this paper integrates both fixed and variable costs of implementing blockchain to explore the dual-cost thresholds at which the target entity or entities are willing to implement blockchain in green agricultural supply chains.

### 2.4. Research gaps

To systematically illustrate the distinctiveness of this paper, a comparison with the most highly related literature is conducted in detail, which is presented in Table 2. Although applying blockchain technology to the agricultural industry has been rapidly increasing, only a small amount of literature such as [31] simultaneously investigated the performance improvement and transparency enhancement from implementing blockchain in green agricultural supply chains, resulting in a significant inconsistency between academic theorization and practical exploration. Therefore, this paper focuses on the specific field of green agricultural supply chains instead of the broad agricultural industry.

**Table 2:** Comparisons between typical highly related studies and this paper.

Typical literature	Roles		Entities				Costs		Topics	
	performance	transparency	single	multiple	given	voluntary	fixed	variable	willingness	agricultural
[3]		√	√		√		√			
[32]	√			√	√		√	√		
[5]	√	√	√		√		√			
[15]	√		√		√			√		√
[23]		√	√	√	√		√		√	
[33]	√		√		√		√	√		
This paper	√	√	√	√		√	√	√	√	√

The contributions of this paper are reflected in the following three points.

First, this paper models supply chain entities’ endogenous blockchain adoption decisions based on their economic inclination, examining three scenarios: independent adoption by manufacturers, independent adoption by retailers, and joint cooperative adoption. Existing literature mostly treats blockchain implementers—whether a single member or multiple members—as exogenously predetermined, rather than stemming from their economic intentions. Such studies fail to capture economic willingness and overlook supply chain firms’ voluntary internal motivation. The novelty of this work lies in endogenizing manufacturers’ and retailers’ adoption behavior linked to economic inclination, rather than treating implementers as an exogenous premise. This fills the literature gap by capturing the internal intentions and economic drivers behind firms’ voluntary blockchain adoption.

Second, this paper concurrently investigates both fixed costs and variable costs of implementing blockchain to explore the dual-cost thresholds at which the designated participant or participants are willing to implement blockchain in green agricultural supply chains. However, previous literature either examines only one dimension of the fixed and variable blockchain implementation costs to derive a single-cost condition under which the implementer can achieve positive profits, or fails to identify the interactive dual-cost thresholds favorable to implementers, thereby always ignoring the interplay between the economic willingness of each side and the resulting stable equilibria, even when both cost dimensions are incorporated simultaneously.

Third, this paper explores optimal equilibria (such as product greenness) via a global optimization approach, where suitable participants are selected to implement blockchain. However, previous literature has only probed optimal equilibria through a local optimization approach, where designated blockchain implementers are fixed and exogenous.

## 3. Description of problem and assumptions

### 3.1. Framework

This paper explores a green agricultural supply chain consisting of a manufacturer and a retailer, who are located in the upstream and downstream, respectively. The manufacturer and the retailer plan to implement blockchain in the supply chain system independently or jointly under respective suitable conditions, or even refuse blockchain if conditions are unacceptable. No matter by whom and no matter how blockchain technology is implemented, there always exist fixed costs which may be shared between them and variable costs which must be borne on one’s own individually. According to the relevant conditions, there are four possible cases: none deploy blockchain (Model A), the manufacturer implements blockchain independently (Model M), the retailer implements blockchain independently (Model R), and both the manufacturer and the retailer implement blockchain jointly (Model MR), which occurs in practice contextually. In each case, prior studies always compulsorily designate the entity or entities implementing blockchain without considering whether they are willing to do so. To make up for this shortcoming, this paper investigates the economic willingness, and furthermore examines who should be selected to implement blockchain in each scenario specified by dual-cost thresholds at which supply chain entities are willing to do so.

The concrete steps of the framework include the following:

**Step 1.** *By solving the equilibria of each model, the optimal profits that the manufacturer and the retailer can earn in each case are obtained, respectively.*

**Step 2.** *By comparing the profits in each case, the economic willingness of each entity is specified with dual-cost thresholds.*

Either the manufacturer or the retailer could implement blockchain initiatively when their partners have not, or reactively when their partners have done so. The dual costs refer to the fixed deployment costs and variable operational costs of implementing blockchain.

**Step 3.** *By integrating and classifying threshold-based scenarios according to dual-cost thresholds, the optimal entity selection for implementing blockchain in each scenario is determined.*

There is a qualitative breakthrough in the research paradigm from Step 1 to Step 3, which reflects the original contribution of this paper. In each model defined in Step 1, supply chain entities passively accept mandatory implementation without considering their economic willingness, which is the assumption commonly made in prior studies. In each scenario defined in Step 3, on the contrary, supply chain entities actively choose to implement blockchain driven by their intrinsic incentives, which extends the boundary of previous literature

### 3.2. Assumptions

As the common approach to simplify the problem in previous literature, some assumptions are configured as follows.

**Assumption 1.** *Entities in the green agricultural supply chain.*

The supply chain system includes a manufacturer and a retailer, who are located in the upstream and downstream and denoted as m and r respectively. In a green production approach, the manufacturer plants crops, processes them into standard packaged agricultural products, and sells them to the retailer at the wholesale price w, where the unit cost borne by the manufacturer is c. Then, the retailer distributes and sells them to final consumers at the retail price p. According to the widely used method in previous research such as [39], all processing, packaging, refrigeration, transportation, and other expenses are omitted, which is useful for concentrating on the key questions. Required by consumers’ green preferences, the manufacturer wants to improve the greenness of agricultural products by adopting green technology. As configured in existing research such as [40], the manufacturer has to bear the investment costs $C\left(e\right)=\frac{\text{1}}{\text{2}}ke^{\text{2}}$, in which the marginal coefficient k and the green level e are abbreviated as the marginal green coefficient and product greenness, respectively.

**Assumption 2.** *Distrust in product greenness information.*

Product greenness information may be attenuated, exaggerated, or misrepresented during transmission. Every link and node in the long journey of agricultural products from field to table, spanning both time and distance, can affect their greenness [1]. Moreover, this may lead to greenwashing behavior; that is, companies may intentionally release false information about product greenness. For example, Hangzhou West Lake Longjing tea is in significant market demand, but its core production areas are limited. Some unscrupulous merchants produce flat green tea and market it as Longjing tea, which makes it difficult for ordinary consumers to distinguish authentic products from counterfeits. Due to this kind of potential transmission distortion, consumers do not fully trust the information asserted by manufacturers, and take it with a grain of salt. Regarding the claimed product greenness e, consumers view it with skepticism (1−ξ)e. The vagueness of green information ξ, similar to the information traceability level discussed in [3], but contrary to the trust degree defined by [41], indicates consumers’ doubts about the product greenness.

**Assumption 3.** *Costs of implementing blockchain.*

The costs of implementing blockchain can be classified into deployment costs and operational costs, which is common in extant literature such as [15] and [40]. When implementing blockchain technology, the green agricultural supply chain incurs the fixed deployment costs f and variable operational costs Dv, where D represents the market demand equaling the production quantity at equilibrium, and v represents the unit operational costs. The fixed deployment costs refer to the capital expenditures required for building a blockchain system, including but not limited to on-site environmental monitoring terminals, data transmission infrastructure such as industrial communication gateways, and blockchain node storage servers. The variable operational costs refer to the ongoing expenses incurred from collecting data on the planting and breeding environment, growth cycle, seedling sources, climate, cultivation, receipt and shipment quantities, product batches, and other aspects of agricultural products, as well as daily data entry labor costs and real-time data cloud access fees uploading such data to the blockchain system. This baseline model focuses only on the above fixed deployment costs and variable operational costs, adopting reasonable simplifications to preserve analytical tractability. Although behavioral factors including organizational dynamics, supply chain power asymmetries and regulatory constraints are not explicitly incorporated into the current framework, the results remain robust to some extent. From the perspective of behavioral economics, blockchain adoption decisions are determined by the trade-off between utility arising from behavioral constraints and economic costs, whereby behavioral factors and economic costs are logically consistent in essence. Whether behavioral factors are taken into account or not, the research conclusions share the same overall trend and evolutionary direction, differing merely in the degree of variation. Accordingly, this parsimonious modeling specification is widely adopted in extant literature, as documented in [15] and [40], which is advantageous for centering on the core incentives underlying blockchain implementation while streamlining the game-theoretic analysis of supply chain interactions.

In unilateral blockchain implementation, the corresponding single entity bears all the blockchain deployment and operational costs exclusively. Under joint implementation, the corresponding multiple entities bear their own operational costs and share deployment costs proportionally [3], and deployment costs are furthermore assumed to be shared equally to simplify the model when ensuring its effectiveness [5]. Such equal sharing of fixed deployment costs in the joint-implementation case does not bias the results. First, existing research generally posited that the expenses incurred in blockchain technology deployment within supply chains were distributed across multiple supply chain participants [5], where each party undertaken a specific portion of the overall fixed costs [33]. Second, the equal cost-sharing setup adopted in this study aligns with standard modeling approaches in prior literature. For example, [5] treated the cost allocation proportion as an exogenous variable, and their framework inherently accommodated the scenario of identical cost sharing as a special instance. Third, this simplifying assumption will not compromise the generalizability of the derived results. Consequently, the equal cost sharing merely is taken to streamline the analysis and concentrate on core research topics, namely participants’ economic intentions and entity selection strategies. If designating the sharing ratio as an exogenous parameter, as in [5] the mathematical model would become unnecessarily intricate, yet the core research outcomes would remain unchanged.

**Assumption 4.** *Ways of implementing blockchain.*

Previous literature has rudimentarily identified individual implementations such as in [21] and cooperative implementations such as in [31]. This paper classifies blockchain implementation ways based on two criteria: economic willingness and behavioral outcome.

On the criterion of economic willingness, both the manufacturer and retailer can autonomously choose between initiative implementation and reactive implementation. In terms of initiative willingness, the entity proactively implements blockchain independently regardless of its partner’s behavior, even when its partner has not adopted blockchain technology. In terms of reactive willingness, the entity chooses follow-up implementation only if its partner has already done so.

On the criterion of behavioral outcome, both the manufacturer and the retailer implement blockchain either independently or jointly. In independent implementation, the entity implements blockchain solely and thus bears both the deployment and operational costs while its partner does not participate. In joint implementation, the entities implement blockchain together and thus share the deployment costs proportionally and bear own operational costs individually.

Economic willingness determines behavioral outcomes. For example, if both parties prefer initiative willingness, the outcome must be that only one, which is selected randomly, implements blockchain independently; if both parties prefer reactive willingness, the outcome must be that both implement blockchain jointly; if one prefers initiative willingness but the other prefers reactive willingness, the outcome must be that neither implements it at all.

**Assumption 5.** *Influences of blockchain on market demand.*

Implementing blockchain can eliminate the information distrust about product greenness. All information, including but not limited to product greenness information, cannot be revised once it is uploaded and stored in the blockchain platform system. Consumers could trace every node in the circulation of agricultural products, which ensures the product greenness information remains always true and accurate, namely ξ=0. Numerous existing studies such as [21,22], and [36], have adopted the similar full transparency setting, which presume that blockchain deployment enables consumers to fully trust product-related information. In this way, blockchain implementation can expand the market demand, which could be configured as $D_{N}=a-\alpha p+\beta (1-\xi )e$ and $D_{B}=a-\alpha p+\beta e$ before and after implementing blockchain respectively, where a represents the potential market demand, while α and β represent price sensitivity coefficient and green sensitivity coefficient, respectively.

Actually, such assumption of perfect transparency is general because it is equivalent to that of partial transparency widely adopted to describe how blockchain implementation affects market demand in previous research such as [3] and [39]. On the contrary, in the case of partial transparency with $0<\xi^{\prime }<\xi$, the corresponding market demand with implemented blockchain becomes $D\prime_{B}=a-\alpha p+\beta (1-\xi^{\prime })e$. Let $\beta^{\prime }=\beta (1-\xi^{\prime })$, then $\beta =\frac{\beta^{\prime }}{1-\xi^{\prime }}$. On one hand, substituting $\beta =\frac{\beta^{\prime }}{1-\xi^{\prime }}$ into $D_{N}=a-\alpha p+\beta (1-\xi )e$, we yield $D\prime_{N}=a-\alpha p+\beta^{\prime }\frac{1-\xi }{1-\xi^{\prime }}e$ $=a-\alpha p+\beta^{\prime }\left(1-\frac{\xi -\xi^{\prime }}{1-\xi^{\prime }}\right)e$. Moreover, let $\xi^{\prime \prime }=\frac{\xi -\xi^{\prime }}{1-\xi^{\prime }}$, then $D\prime_{N}=a-\alpha p+\beta^{\prime }\left(1-\xi^{\prime \prime }\right)e$. On the other hand, substituting $\beta =\frac{\beta^{\prime }}{1-\xi^{\prime }}$ into $D\prime_{B}=a-\alpha p+\beta (1-\xi^{\prime })e$, we can yield $D\prime_{B}=a-\alpha p+\beta^{\prime }e$. It is clear that $D\prime_{N}=a-\alpha p+\beta^{\prime }\left(1-\xi^{\prime \prime }\right)e$ and $D\prime_{B}=a-\alpha p+\beta^{\prime }e$ under the assumption of partial transparency actually are tantamount to $D_{N}=a-\alpha p+\beta (1-\xi )e$ and $D_{B}=a-\alpha p+\beta e$ under the assumption of perfect transparency; the only difference lies in their symbolic forms. Thus, the perfect transparency assumption is general and essentially equivalent to the partial transparency case. The perfect transparency assumption not only simplifies the mathematical derivation, but also preserves the generalizability of the results.

**Assumption 6.** *Reputational gains from blockchain implementation.*

Implementing blockchain technology can promote information transparency and traceability, as well as record and accumulate positive transaction credits, thereby leading to positive impacts such as expanding orders and potential cooperation opportunities. This generates additional revenue, defined as blockchain-based reputational gain. For example, a report by Ant Blockchain Technology shows that Wuchang Rice has established a traceability system featuring three confirmations, one inspection, and one code to combat counterfeiting. By setting up traceability labels with a Quick Response code, the end-to-end information from the field to the dining table is recorded and made traceable. This has resulted in a win-win situation of increasing farmer income, improving operational efficiency, enhancing market credibility, and creating a brand with a value of ¥ 66.793 billion. The larger the blockchain system, the wider the regions it covers, and the more technical nodes it endows. In other words, the larger the blockchain system, the higher the fixed deployment costs and the more blockchain-based reputational gains it generates. Once the blockchain technology is implemented, the distrust in product greenness is eliminated and thus the demand for the entire supply chain system is boosted. So, the reputational gains come from the elimination of distrust in product greenness, which accrue to all entities along the supply chain [5] and are irrelevant to who implements blockchain.

Theoretically, there is sufficient justification. Some literature such as [42] makes a similar assumption of spillover effects, where the blockchain R&D may have positive spillover effects on other companies and the external effects of a food company’s R&D activities lowers the unit production costs of its competitors. Empirically, there are industrial practices supporting such an assumption. For instance, after implementing blockchain technology, Dang-Shan Crispy Pear achieved an all-round breakthrough. It sold 300,000 pieces within three days, generating ¥4.5 million in sales, a 200% year-on-year improvement. This also benefited related sales platforms like TikTok and Tmall Supermarket, even though they did not participate in the blockchain implementation for the Crispy Pear supply chain at all. Additionally, in the view of consumers, only if the supply chain system adopts blockchain technology will they trust the greenness of agricultural products deeper and be willing to buy more at a higher price. They neither know nor care who implements blockchain. Thus, every supply chain entity, not only those implementing blockchain actively but also those free riders who do not actually implement it, can attain the reputational gains.

Therefore, similar to the approach of spillover effect in [42], which differentiates reputational gains for active implementers and those for free riders, the reputational gains for active implementers are denoted as $u=\frac{\text{1}}{\text{2}}gf^{\text{2}}$, while those of free riders are denoted as $\frac{\text{1}}{\text{2}}u$, where the coefficient of marginal reputation is represented by g, and f denotes the fixed deployment costs of implementing blockchain. Such a functional form accurately describes the characteristics of high-speed, accelerating growth at the current stage of blockchain technology implementation in green agricultural supply chains in China.

**Assumption 7.** *Parameter constraints.*

To simplify the model while keeping its effectiveness with positive equilibria, the parameters are assumed to meet the constraints as follows. The fixed deployment costs are high enough to meet $f>\frac{\text{2}}{g}$, while the variable operational costs are low enough to satisfy $b<\frac{a-2\alpha c}{4\alpha }$. To assess their relative magnitudes, we refrain from a direct comparison between deployment and operational costs, as these two cost types often differ substantially in scale. Instead, consistent with the standard methodology adopted in prior studies [43], we evaluate operational costs relative to a specified proportion of deployment costs. Additionally, the marginal coefficient of green investment is constrained to $k>\frac{\beta^{\text{2}}}{4\alpha }$, while the potentialmarket demand is assumed to satisfy the restriction $a>\frac{2ck\alpha^{\text{2}}+\sqrt{3\alpha f\left(gf-2\right){\left(4\alpha k-\beta^{\text{2}}\right)}^{\text{2}}}}{2\alpha k}$. Some similar constraints are documented in existing literature such as [3] and [28], and [42], which ensure the existence of unique optimal and non-negative equilibrium solutions for these models and avoid trivial results. Moreover, the above condition on market demand$a>\frac{2ck\alpha^{\text{2}}+\sqrt{3\alpha f\left(gf-2\right){\left(4\alpha k-\beta^{\text{2}}\right)}^{\text{2}}}}{2\alpha k}$ with the restriction $f>\frac{\text{2}}{g}$ equals $\frac{\text{2}}{g}<f<\frac{1+\sqrt{\frac{4g\alpha^{\text{2}}k^{\text{2}}(a-c\alpha {)}^{\text{2}}}{3\alpha {\left(4\alpha k-\beta^{\text{2}}\right)}^{\text{2}}}+1}}{g}$, which avoids an excessively high deployment cost and thus prevents the potential problem of over-investment.

### 3.3. Notations

The used notations with their explanations are illustrated in Table 3.

**Table 3:** Model notations with explanations.

Symbols		Explanations
subscripts	m	upstream manufacturer
	r	downstream retailer
superscripts	A	absent implementation
	M	manufacturer-independent implementation
	R	retailer-independent implementation
	MR	manufacturer-retailer joint implementation
decision variables	w	wholesale price
	e	product greenness
	p	retail price
parameters	c	unit production cost
	v	variable operational costs of implementing blockchain
	f	fixed deployment costs of implementing blockchain
	u	blockchain-based reputational gains
	g	marginal blockchain reputation
	a	potential market demand
	α	price sensitivity
	β	greenness sensitivity
	k	marginal coefficient of green investment
	ξ	vagueness of green information
	π	supply chain profits
	$D_{N}$	demand before implementing blockchain
	$D_{B}$	demand after implementing blockchain
	${\overset{―}{b}}_{\text{1}}$	threshold for retailer’s initiative willingness
	${\overset{―}{b}}_{\text{2}}$	threshold for manufacturer’s initiative willingness
	${\overset{―}{b}}_{\text{3}}$	threshold for retailer’s reactive willingness
	${\overset{―}{b}}_{\text{4}}$	threshold for manufacturer’s reactive willingness
	$\Omega_{\text{1}}$	scenario with extremely high operational costs
	$\Omega_{\text{2}}$	scenario with moderately high operational costs
	$\Omega_{\text{3}}$	scenario with balanced deployment and operational costs
	$\Omega_{\text{4}}$	scenario with moderately high deployment costs
	$\Omega_{\text{5}}$	scenario with extremely high deployment costs

## 4. Model construction

As the basis of exploring each entity’s economic willingness to implement blockchain, this section examines the price and product greenness decisions of the green agricultural supply chain with different blockchain implementing entities. Employing the usual analytical framework in prior studies such as [23] and [27], the model is categorized into four cases: Model A where none implements blockchain, Model M where the upstream manufacturer implements blockchain independently, Model R where the downstream retailer implements blockchain independently, and Model MR where both the upstream manufacturer and the downstream retailer implement blockchain jointly. In each case, as is common in previous literature, the implementer or implementers are designated mandatorily, whose economic willingness has been ignored and thus will accordingly be explored in the next section.

All cases follow the same game sequence. At the first stage, the upstream manufacturer simultaneously determines the product greenness and the wholesale price. At the second stage, the downstream retailer sets the retail price. The method of backward induction is adopted to derive the optimal price and product greenness solutions for each case.

### 4.1. Model A: Absent implementation

In the case of Model A, no entity implements blockchain technology at all, and thus the greenness information may be misrepresented in the course of transmission. Consumers partially distrust the claimed information of product greenness e. The market demand could be formulated as $D_{N}=a-\alpha p+\beta (1-\xi )e$, where ξ captures the degree to which the information is disbelieved. The profits of the manufacturer are expressed as $\overset{A}{\underset{m}{\pi }}=(w-c)D_{N}-\frac{\text{1}}{\text{2}}ke^{\text{2}}$, while the retailer’s profits are denoted as $\overset{A}{\underset{r}{\pi }}=(p-w)D_{N}$. Thus, by reasoning backward, the optimal price and product greenness are listed in Theorem 1. Proofs of all Theorems and Lemmas are provided in the Supporting Information.

**Theorem 1.** *In the green agricultural supply chain without blockchain implementation, the optimal greennessis denoted as* $e^{A*}=\frac{\beta (1-\xi )(a-\alpha c)}{4\alpha k-\beta^{\text{2}}(1-\xi {)}^{\text{2}}}$*. The optimal wholesale price is* $w^{A*}=\frac{c\left(2\alpha k-\beta^{\text{2}}{\left(1-\xi \right)}^{\text{2}}\right)+2ak}{4\alpha k-\beta^{\text{2}}(1-\xi {)}^{\text{2}}}$*, while the retail price is*$p^{A*}=\frac{c\left(\alpha k-\beta^{\text{2}}{\left(1-\xi \right)}^{\text{2}}\right)+3ak}{4\alpha k-\beta^{\text{2}}(1-\xi {)}^{\text{2}}}$*. The corresponding profits of the manufacturer are* $\overset{A*}{\underset{m}{\pi }}=\frac{k(a-\alpha c{)}^{\text{2}}}{8\alpha k-2\beta^{\text{2}}(1-\xi {)}^{\text{2}}}$*, and those of the retailer are* $\overset{A*}{\underset{r}{\pi }}=\frac{\alpha k^{\text{2}}(a-\alpha c{)}^{\text{2}}}{{\left(4\alpha k-(1-\xi {)}^{\text{2}}\beta^{\text{2}}\right)}^{\text{2}}}$*.*

In the case of absent blockchain, from the results presented in Theorem 1, the effects of information vagueness on the equilibria are drawn in Corollary 1.

**Corollary 1.** $\frac{\partial e^{A*}}{\partial \xi }<0$*,* $\frac{\partial p^{A*}}{\partial \xi }<0$*,* $\frac{\partial \overset{A*}{\underset{m}{\pi }}}{\partial \xi }<0$*, and* $\frac{\partial \overset{A*}{\underset{r}{\pi }}}{\partial \xi }<0$*.*

The information vagueness stemming from consumers’ distrust decreases the greenness and price of agricultural products, as well as the profits of the manufacturer and retailer. If not deployed, greenness information is likely to be distorted. Consequently, consumers disbelieve the greenness information, leading to a decline in purchase behavior regarding both the price (quality aspect) and quantity (amount aspect). The contraction in market demand will decrease the profits of both the manufacturer and the retailer. Subsequently, the manufacturer has to reduce green investment, which in turn triggers a further decline in greenness and purchasing. Therefore, it is highly necessary to implement blockchain to eliminate the negative effect of greenness information distrust.

### 4.2. Model M: manufacturer-independent implementation

In the case of Model M, the manufacturer independently deploys blockchain and undertakes both the deployment costs and operational costs in the green agricultural supply chain. The product greenness information is uploaded to the blockchain system and is transmitted precisely. For example, China Green Foods Group, as a leading manufacturer of agricultural products, collaborates with China Securities Tech, a blockchain service provider, in the field of blockchain-based agricultural product traceability. Implementing blockchain and bearing both the deployment costs and operational costs, China Green Foods Group uploads the full lifecycle data, from planting to harvesting and from storage to sale, to the blockchain platform system. In this way, the green agricultural supply chain can be traced, agricultural resources can be precisely managed, and the quality of crops and agricultural products can be monitored in real time.

When the manufacturer implements blockchain independently, the distrust of consumers in the greenness information is eliminated. Then, the market demand becomes $D_{B}=a-\alpha p+\beta e$. The manufacturer, as the blockchain implementer, obtains the blockchain-based reputational gains $u=\frac{\text{1}}{\text{2}}gf^{\text{2}}$, while the retailer, as the entity free-riding on blockchain implementation, only attains $\frac{\text{1}}{\text{2}}u=\frac{\text{1}}{\text{4}}gf^{\text{2}}$. The profits of the manufacturer are $\overset{M}{\underset{m}{\pi }}=(w-c-v)D_{B}+u-\frac{\text{1}}{\text{2}}ke^{\text{2}}-f$, and those of the retailer are $\overset{M}{\underset{r}{\pi }}=(p-w)D_{B}+\frac{u}{\text{2}}$. By the approach of backward reasoning, the optimal price and product greenness are presented in Theorem 2.

**Theorem 2**. *In the green agricultural supply chain with independently implemented blockchain by the manufacturer, theoptimal greenness is* $e^{M*}=\frac{\beta \left(a-\alpha \left(c+b\right)\right)}{4\alpha k-\beta^{\text{2}}}$*, and the optimal wholesale price is* $w^{M*}=\frac{(c+v)\left(2\alpha k-\beta^{\text{2}}\right)+2ak}{4\alpha k-\beta^{\text{2}}}$*, while the optimal retailprice is* $p^{M*}=\frac{(c+v)\left(\alpha k-\beta^{\text{2}}\right)+3ak}{4\alpha k-\beta^{\text{2}}}$*. Correspondingly, the profits of the upstream manufacturer are* $\overset{M*}{\underset{m}{\pi }}=\frac{k{\left(a-(c+v)\right)}^{\text{2}}+f(gf-2)(4\alpha k-\beta^{\text{2}})}{2\left(4\alpha k-\beta^{\text{2}}\right)}$*, while those of the downstream retailer are* $\overset{M*}{\underset{r}{\pi }}=\frac{4k^{\text{2}}\alpha^{\text{3}}(c+v{)}^{\text{2}}+4\alpha ak^{\text{2}}\left(a-2\alpha \left(c+v\right)\right)+gf^{\text{2}}\left(8\alpha k\left(2\alpha k-\beta^{\text{2}}\right)+\beta^{\text{4}}\right)}{4{\left(4\alpha k-\beta^{\text{2}}\right)}^{\text{2}}}$*.*

### 4.3. Model R: retailer-independent implementation

In the case of Model R, the retailer deploys blockchain independently and undertakes all the deployment costs and operational costs in the green agricultural supply chain. For example, Yong-Hui Superstores, a leading superstore corporation of China, sells blockchain-based Turbots, where the blockchain technology of traceability is utilized to record the full lifecycle data from fry cultivation, aquaculture to store sales. The product information is automatically uploaded to the blockchain system and is transmitted precisely. Every piece of Turbot is assigned a code for traceability, which covers and reflects all-course information such as those of production and quarantine inspection.

When the retailer implements blockchain independently, the distrust of consumers in the greenness information is eliminated too. The market demand also becomes $D_{B}=a-\alpha p+\beta e$, which has nothing to do with who implements the blockchain. The retailer, as the blockchain implementer, obtains the blockchain-based reputational gains $u=\frac{\text{1}}{\text{2}}gf^{\text{2}}$, while the manufacturer, as the free-rider in blockchain implementation, only attains $\frac{\text{1}}{\text{2}}u=\frac{\text{1}}{\text{4}}gf^{\text{2}}$. Then, the profits of the manufacturer are denoted as $\overset{R}{\underset{m}{\pi }}=(w-c)D_{B}+\frac{u}{\text{2}}-\frac{\text{1}}{\text{2}}ke^{\text{2}}$, and those of the retailer represented by $\overset{R}{\underset{r}{\pi }}=(p-w-v)D_{B}+u-c_{f}$. Similarly, by backward reasoning, the optimal price and product greenness are illustrated in Theorem 3.

**Theorem 3.** *In the green agricultural supply chain with independently implemented blockchain by the retailer, theoptimal greenness is* $e^{R*}=\frac{\beta \left(a-\alpha \left(c+v\right)\right)}{4\alpha k-\beta^{\text{2}}}$*, the optimal wholesale price is* $w^{R*}=\frac{c\left(2\alpha k-\beta^{\text{2}}\right)+2k\left(a-\alpha v\right)}{4\alpha k-\beta^{\text{2}}}$*, and thecorresponding optimal retail price is* $p^{R*}=\frac{\left(c+v\right)\left(\alpha k-\beta^{\text{2}}\right)+3ak}{4\alpha k-\beta^{\text{2}}}$*. Accordingly, the optimal profits of the manufacturer are*$\overset{R*}{\underset{m}{\pi }}=\frac{gf^{\text{2}}\left(4\alpha k-\beta^{\text{2}}\right)+2k\alpha^{\text{2}}{\left(c+v\right)}^{\text{2}}+2ak\left(a-2\alpha \left(c+v\right)\right)}{4\left(4\alpha k-\beta^{\text{2}}\right)}$*, and those of the retailer are* $\overset{R*}{\underset{r}{\pi }}=\frac{2k^{\text{2}}\alpha^{\text{3}}{\left(c+v\right)}^{\text{2}}+f(gf-2)\left(8\alpha k\left(2\alpha k-\beta^{\text{2}}\right)+\beta^{\text{4}}\right)+2\alpha ak^{\text{2}}\left(a-2\alpha \left(c+v\right)\right)}{2{\left(4\alpha k-\beta^{\text{2}}\right)}^{\text{2}}}$*.*

### 4.4. Model MR: manufacturer-retailer joint implementation

In the case of Model MR, the manufacturer and the retailer jointly implement blockchain, thus sharing equally the fixed deployment costs and paying their own variable operational costs in the green agricultural supply chain. Product information is submitted to the blockchain system in real time and transmitted precisely, ensuring the traceability of crop quality. For instance, the Wuchang Farmer Cooperative has conducted comprehensive cooperation with Tmall and Cai-Niao Logistics under Alibaba Group by introducing blockchain traceability technology into the supply chain of Wuchang rice. With the support of the blockchain platform system, the identity of Wuchang rice can be traced back to the planting stage. Each bag of rice sold in the Tmall Flagship Store of Wuchang Rice has an exclusive unique Quick Response Code. By opening Alipay and scanning this unique Quick Response Code, consumers can access the detailed traceability information, ranging from the specific growing location, seed types, and fertilizers applied to the entire logistics process.

The joint blockchain implementation eliminates the consumers’ distrust in product greenness, and thus the market demand becomes $D_{B}=a-\alpha p+\beta e$. The manufacturer and the retailer, both as blockchain implementers, can obtain own blockchain-based reputational gains $u=\frac{\text{1}}{\text{2}}gf^{\text{2}}$ respectively. Consequently, the profits of the manufacturer are derived as $\overset{MR}{\underset{m}{\pi }}=(w-c-v)D_{B}+u$ $-\frac{\text{1}}{\text{2}}ke^{\text{2}}-\frac{\text{1}}{\text{2}}f$, and those of the retailer are $\overset{MR}{\underset{r}{\pi }}=(p-w-v)D_{B}$ $+u-\frac{\text{1}}{\text{2}}f$. By backward reasoning, the optimal price and product greenness are derived in Theorem 4.

**Theorem 4.** *In the green agricultural supply chain with jointly implemented blockchain, the optimal greenness is*$e^{MR*}=\frac{\beta \left(a-\alpha \left(c+2v\right)\right)}{4\alpha k-\beta^{\text{2}}}$*, the optimal wholesale price and retail price are* $w^{MR*}=\frac{2k(\alpha c+a)-\left(c+v\right)\beta^{\text{2}}}{4\alpha k-\beta^{\text{2}}}$ *and*$p^{MR*}=\frac{\left(c+2v\right)\left(\alpha k-\beta^{\text{2}}\right)+3ak}{4\alpha k-\beta^{\text{2}}}$*, respectively. At the equilibria of interactive game decisions, the corresponding optimal profits ofthe manufacturer are* $\overset{MR*}{\underset{m}{\pi }}=\frac{f(4\alpha k-\beta^{\text{2}})(gf-1)+k\alpha^{\text{2}}{\left(c+2v\right)}^{\text{2}}+ak\left(a-2\alpha \left(c+2v\right)\right)}{2\left(4\alpha k-\beta^{\text{2}}\right)}$*, while those of the retailer are*$\overset{MR*}{\underset{r}{\pi }}=\frac{f\left(gf-1\right)\left(8\alpha k\left(2\alpha k-\beta^{\text{2}}\right)+\beta^{\text{4}}\right)+2\alpha^{\text{3}}k^{\text{2}}{\left(c+2v\right)}^{\text{2}}+2\alpha ak^{\text{2}}\left(a-2\alpha \left(c+2v\right)\right)}{2{\left(4\alpha k-\beta^{\text{2}}\right)}^{\text{2}}}$*.*

From the above Theorems 2, 3, and 4, the effects of the variable operational costs of implementing blockchain on the product greenness, price, and profits of the green agricultural supply chain can be derived as listed in the following corollaries 2 and 3.

**Corollary 2.** $e^{R*}=e^{M*}>e^{MR*}>e^{N*}$, $p^{MR*}>p^{R*}=p^{M*}>p^{N*}$, $q^{MR*}<q^{R*}=q^{M*}<q^{N*}$.

First, the implementation of blockchain always improves the greenness of agricultural products, regardless of which entity implements it. However, compared with independent implementation by a single entity, joint implementation by two entities will diminish the product greenness. The independent implementation by a single entity has already eradicated consumers’ distrust in product greenness. In contrast, joint implementation by two entities does not bring any marginal improvements to trust in green information, but only generates additional costs. These additional costs will lead to a reduction in green investment, thereby lowering product greenness.

Second, the implementation of blockchain always raises the price of agricultural products. Whether an entity implements blockchain independently or jointly with other participants, the implementation costs will inevitably be passed down the agricultural supply chain link by link, driving up the prices borne by end consumers.

Third, the implementation of blockchain always reduces the demand for agricultural products, regardless of which entity implements it or what the method is adopted. Based on the first point above, the implementation of blockchain improves the greenness of agricultural products, which in turn allows consumers to obtain higher utility from a smaller quantity, thereby reducing demand. According to the second point above, the implementation of blockchain drives up prices, which also leads to a reduction in demand. Consequently, the implementation of blockchain results in an overall reduction in demand.

**Corollary 3.** *(1)* $\frac{\partial e^{R*}}{\partial b}<0$*,* $\frac{\partial e^{M*}}{\partial b}<0$*,* $\frac{\partial e^{MR*}}{\partial b}<0$*. (2)* $\frac{\partial p^{R*}}{\partial b}>0$*,* $\frac{\partial p^{M*}}{\partial b}>0$*,* $\frac{\partial p^{MR*}}{\partial b}>0$*. (3)* $\frac{\partial \overset{R*}{\underset{m}{\pi }}}{\partial b}<0$*,* $\frac{\partial \overset{M*}{\underset{m}{\pi }}}{\partial b}<0$*,* $\frac{\partial \overset{MR*}{\underset{m}{\pi }}}{\partial b}<0$;$\frac{\partial \overset{R*}{\underset{r}{\pi }}}{\partial b}<0$*,* $\frac{\partial \overset{M*}{\underset{r}{\pi }}}{\partial b}<0$*,* $\frac{\partial \overset{MR*}{\underset{r}{\pi }}}{\partial b}<0$*.*

First, irrespective of whether blockchain is implemented independently or jointly, its operational costs may harm product greenness. Although blockchain implementation could remove consumers’ distrust in the greenness information, it is possible that such implementation may instead lead to a reduction in the product greenness because the implementation costs may crowd out and thus reduce green investment. Therefore, the implementation of blockchain does not guarantee a rise in the greenness of agricultural products, which is inconsistent with conventional understanding.

Second, irrespective of whether blockchain is implemented independently or jointly, its operational costs push up agricultural product prices. On one hand, these costs will be passed along through each link and node of the green agricultural supply chain and eventually transferred to consumers, who have to pay higher prices. On the other hand, when blockchain adoption eliminates the distrust in the greenness information, consumers are willing to pay higher prices for self-perceived greener agricultural products.

Third, irrespective of whether blockchain is deployed independently or jointly, its operational costs diminish the profits of every entity in the green agricultural supply chain. Adopting blockchain may generate both positive and negative impacts. The positive impact arises from expanded market demand due to the elimination of consumers’ distrust. The negative impact includes direct implementation costs and indirect shrinkage of market demand, the latter is caused by decreased green investment, which is crowded out by blockchain investment. It is possible that the negative impacts outweigh the positive ones, and thus no entity is willing to implement blockchain, which deviates from the general consensus too. For this very reason, investigating the economic willingness of different stakeholders to implement blockchain is essentially important in the green agricultural supply chain.

### 4.5. Game matrix for blockchain implementation

**Fig 1:**
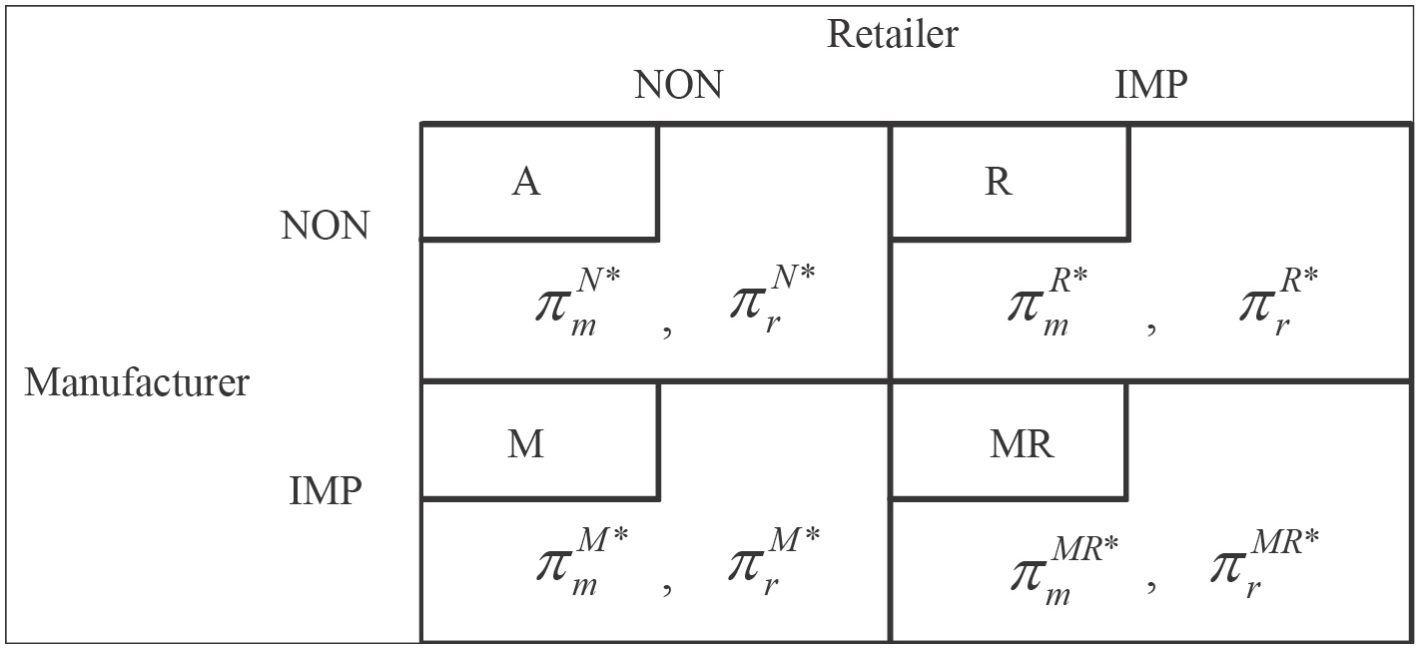
Game matrix for blockchain implementation.

Summarizing the above four models, the game matrix for blockchain deployment can be illustrated as listed in Fig 1, where both the upstream manufacturer and the downstream retailer have two actions: the strategy of implementation denoted as IMP and the option of non-implementation denoted as NON. [44] constructed a similar game matrix in the context of shipping supply chains.

The four strategy profiles in the game matrix correspond one-to-one with the above four models. The payoffs in the game matrix align exactly with the profits presented in Theorems 1–4. For example, the strategy profile where the manufacturer chooses to implement blockchain while the retailer chooses non-implementation corresponds to the Model M, while Model MR corresponds to the strategy profile wherein both the manufacturer and the retailer choose to implement blockchain jointly.

## 5. Economic willingness to implement blockchain

This section examines the economic willingness of supply chain entities to adopt blockchain technology within the framework of a game-theoretic matrix. Taking a typical strategy profile as an example, if the manufacturer opts to implement blockchain while the retailer chooses not to, this outcome constitutes a Nash equilibrium—indicating that the manufacturer is economically willing to act unilaterally, whereas the retailer is not. Broadly, both parties exhibit two distinct types of economic willingness: initiative and reactive. Under initiative willingness, an entity prefers to independently implement blockchain even if its partner does not participate, thereby bearing the full deployment and operational costs alone. In contrast, under reactive willingness, an entity agrees to implement blockchain only when its partner has already done so, allowing the two parties to share deployment costs equally while each still covers its own operational expenses. The specific ceiling thresholds below which the manufacturer or retailer are willing to implement blockchain—either independently or jointly—are analyzed and captured in the following subsections.

### 5.1. Retailer’s initiative willingness

The initiative willingness of the retailer to implement blockchain solely implies that the retailer still chooses to implement blockchain even if the manufacturer has not. It requires that, under the condition that the manufacturer has not implemented it, the retailer can acquire more profits by choosing the action of implementation than choosing the action of non-implementation, that is, $\overset{R*}{\underset{r}{\pi }}>\overset{A*}{\underset{r}{\pi }}$. Such requirement yields the following lemma.

**Lemma 1.** *The retailer is willing to implement blockchain initiatively if and only if the variable operational costs are lower than the first ceiling threshold expressed in terms of deployment costs, namely,* $v<{\overset{―}{b}}_{\text{1}}\left(f\right)$*.*

Here, ${\overset{―}{b}}_{\text{1}}\left(f\right)=\frac{Ak\alpha (a-\alpha c)-\sqrt{\alpha B^{\text{2}}\left(\alpha k^{\text{2}}(a-\alpha c{)}^{\text{2}}-8A^{\text{2}}f\left(gf-2\right)\right)}}{Ak\alpha^{\text{2}}}$ is the first ceiling threshold, where $A=\alpha k-\frac{(1-\xi {)}^{\text{2}}\beta^{\text{2}}}{\text{4}}$, $B=\alpha k-\frac{\beta^{\text{2}}}{\text{4}}$.

Even if the manufacturer has not implemented blockchain, the retailer still has the incentive to initiatively adopt it as long as the operational costs are below the above ceiling threshold. Similarly, [21] defined cost thresholds to capture the conditions for given entities to implement blockchain in low-carbon supply chain.

### 5.2. Manufacturer’s initiative willingness

The initiative willingness of the manufacturer to implement blockchain solely implies that the manufacturer still chooses to implement blockchain even if the retailer has not. It requires that, under the premise that the retailer has not implemented it, the manufacturer can obtain more profits by choosing to implement rather than not to implement, that is, $\overset{M*}{\underset{m}{\pi }}>\overset{A*}{\underset{m}{\pi }}$. Such requirement yields the following lemma.

**Lemma 2.** *The manufacturer is willing to implement blockchain initiatively if and only if the variable operational costs are lower than the second ceiling threshold expressed in terms of deployment costs, namely,* $v<{\overset{―}{b}}_{\text{2}}\left(f\right)$*.*

Here, ${\overset{―}{b}}_{\text{2}}\left(f\right)=\frac{Ak\left(a-\alpha c\right)-\sqrt{ABk\left(k{\left(a-\alpha c\right)}^{\text{2}}-4Af\left(gf-2\right)\right)}}{Ak\alpha }$ is the second ceiling threshold.

Even if the retailer has not implemented blockchain, the manufacturer still finds it profitable to initiatively implement as long as the operational costs are lower than the above ceiling threshold.

From Lemmas 1 and 2, the following corollary is yielded.

**Corollary 4.** *(1)* $\frac{\partial {\overset{―}{b}}_{\text{1}}}{\partial \xi }>0$*,* $\frac{\partial {\overset{―}{b}}_{\text{2}}}{\partial \xi }>0$*. (2)* $\frac{\partial {\overset{―}{b}}_{\text{1}}}{\partial g}>0$*,* $\frac{\partial {\overset{―}{b}}_{\text{2}}}{\partial g}>0$*.*

First, irrespective of who implements blockchain initiatively, the information vagueness raises the ceiling thresholds, and increases the economic willingness of initiative deployment. This reveals the positive effects of information vagueness, which differ from conventional wisdom. The greater the information ambiguity, the more distrust there is in product greenness that blockchain implementation can eliminate, the more demand it can spur, the more incremental profits it can generate, and the higher the operational costs that can be tolerated. Thus, the urgency to implement blockchain becomes more pronounced when facing stronger information vagueness.

Second, irrespective of who implements blockchain initiatively, the blockchain-based reputation raises the ceiling thresholds, and widens the economic willingness of initiative implementation. The more income blockchain technology yields, the higher operational costs that can be tolerated. The trade-off between relevant benefits and costs determines whether to implement blockchain, where the relevant benefits include those from resolving information vagueness and those from technological reputation. Thus, the more prominent the technological reputation is, the stronger the economic willingness of both the manufacturer and the retailer to initiatively adopt blockchain.

### 5.3. Retailer’s reactive willingness

The reactive willingness of the retailer to implement blockchain jointly implies that the retailer follows suit implementing blockchain when the manufacturer has done so. It requires that, under the condition of the manufacturer’s implementation, the retailer can obtain more profits by choosing the action of implementation than choosing the action of non-implementation, that is, $\overset{MR*}{\underset{r}{\pi }}>\overset{M*}{\underset{r}{\pi }}$. Such requirement yields the following lemma.

**Lemma 3-** *The retailer is willing to implement blockchain reactively if and only if the variable operational costs are lower than the third ceiling threshold expressed in terms of deployment costs, namely,* $v<{\overset{―}{b}}_{\text{3}}\left(f\right)$*.*

Here, ${\overset{―}{b}}_{\text{3}}\left(f\right)=\frac{\alpha k\left(a-\alpha c\right)-\sqrt{\alpha \left(\alpha k^{\text{2}}(a-\alpha c{)}^{\text{2}}-12fB^{\text{2}}\left(gf-2\right)\right)}}{3k\alpha^{\text{2}}}$ is the third ceiling threshold.

When the manufacturer has implemented blockchain, the retailer finds it profitable to follow up to implement as long as the operational costs are lower than the above ceiling threshold.

### 5.4. Manufacturer’s reactive willingness

The reactive willingness of the manufacturer to implement blockchain jointly implies that the manufacturer follows suit when the retailer has done so. It requires that, under the premise of the retailer’s implementation, the manufacturer can obtain more profits by choosing the action of implementation than choosing the action of non-implementation, that is, $\overset{MR*}{\underset{m}{\pi }}>\overset{R*}{\underset{m}{\pi }}$. Such requirement yields the following lemma.

**Lemma 4.** *The manufacturer is willing to implement blockchain reactively if and only if the variable operational costs are lower than the fourth ceiling threshold expressed in terms of deployment costs, namely,* $v<{\overset{―}{b}}_{\text{4}}\left(f\right)$*.*

Here, ${\overset{―}{b}}_{\text{2}}\left(f\right)=\frac{Ak\left(a-\alpha c\right)-\sqrt{ABk\left(k{\left(a-\alpha c\right)}^{\text{2}}-4Af\left(gf-2\right)\right)}}{Ak\alpha }$ is the fourth ceiling threshold.

When the retailer has implemented blockchain, the manufacturer finds it profitable to follow up to implement as long as the operational costs are lower than the above ceiling threshold.

From Lemmas 3 and 4, the following corollary is yielded.

**Corollary 5.** *(1)* $\frac{\partial {\overset{―}{b}}_{\text{3}}}{\partial \xi }=0$*,* $\frac{\partial {\overset{―}{b}}_{\text{4}}}{\partial \xi }=0$*. (2)* $\frac{\partial {\overset{―}{b}}_{\text{3}}}{\partial g}>0$*,* $\frac{\partial {\overset{―}{b}}_{\text{4}}}{\partial g}>0$*.*

First, whether the manufacturer or the retailer implements blockchain reactively, the information ambiguity exerts no influence on the ceiling thresholds, nor does it have any impact on their reactive willingness. The case of reactive implementation means that the supply chain partner has implemented blockchain, and thus the distrust in greenness information has already been removed. Therefore, the benefit of reactive implementation stems from the technological reputation rather than the market demand expansion caused by the remove of distrust. When the partner has adopted blockchain, the supply chain entity will become an active implementer if it implements reactively, instead of a free rider if it does not follow up, because the reputational gains of an active implementer are greater than those of a free rider.

Second, whether the manufacturer or the retailer implements blockchain in a passive manner, the blockchain-based reputation lifts the ceiling thresholds, and thus reinforces their reactive willingness. Both the technological reputational gains of an active implementer and those of a free rider grow with the blockchain-based reputation. Then, similar to the situation of initiative implementation, the more prominent the technological reputation is, the higher operational costs that can be borne, and the stronger the economic willingness to implement blockchain reactively.

### 5.5. Comparison and sorting

Lemmas 1–4 illustrate the corresponding ceiling thresholds for which the manufacturer or the retailer is willing to implement blockchain initiatively even if its partner has not done so, or to implement blockchain reactively only if its partner has done so. The comparison and ranking of the above four thresholds are outlined in Lemma 5.

**Lemma 5.** ${\overset{―}{b}}_{\text{4}}\left(f\right)<{\overset{―}{b}}_{\text{3}}\left(f\right)<{\overset{―}{b}}_{\text{2}}\left(f\right)<{\overset{―}{b}}_{\text{1}}\left(f\right)$.

The retailer’s initiative willingness to implement blockchain surpasses the manufacturer’s, which is in turn larger than the reactive willingness of the retailer, and the latter further outweighs the manufacturer’s reactive willingness.

The following two propositions can be drawn by integrating Lemmas 1–5.

**Proposition 1.** *No matter who implements blockchain, the upstream manufacturer or the downstream retailer, the initiative willingness is always stronger than the reactive willingness.*

All supply chain members prefer independent implementation to joint implementation. The player can obtain both the reputational gains and the increment in market revenue resulting from alleviating consumer distrust by implementing blockchain independently, but can acquire only the reputational gains by following up to jointly implement blockchain with its partner. Furthermore, the reputational gains obtained as an active implementer when implementing initiatively are greater than the increment in reputational gains derived from as free-rider to as an implementer when implementing reactively. As a result, the player can gain more revenues by choosing independent implementation than by choosing joint implementation.

Surprisingly, this finding differs substantially from the conventional perception that shareholders should be incentivized to implement blockchain cooperatively. To a certain extent, this displays the properties of the prisoner’s dilemma: cooperation is merely an aspiration, while the equilibrium outcomes locate at independent individual implementation. Consequently, when making policies about agricultural blockchain, governmental policy makers should take into account the economic intentions of supply chain entities and align the economic intentions and extrinsic regulations.

**Proposition 2.** *No matter how blockchain is implemented, initiatively or reactively, the economic willingness of the downstream retailer is always stronger than that of the upstream manufacturer.*

The downstream retailer is always more inclined to implement blockchain than the upstream manufacturer. As the investor cand carrier of green technology, the manufacturer has to shoulder investment costs of green technology. If implementing blockchain independently, the manufacturer has to bear green technology investment costs and both deployment costs and operational costs of blockchain technology; while if implementing blockchain reactively, the manufacturer has to bear investment costs of green technology and only operational costs of blockchain technology. Conversely, if the retailer implements blockchain independently, it only needs to bear both deployment costs and operational costs of blockchain technology; while if it implements blockchain reactively, the retailer only needs to bear its own operational costs of blockchain technology. By comparison, it is clear that no matter whether blockchain is implemented initiatively or reactively, the total costs borne by the retailer are always less than those borne by the manufacturer. Therefore, the thresholds within which the retailer is willing to implement blockchain are higher than those for the manufacturer. In the agricultural product supply chain, there exists a retailer-dominant effect in blockchain implementation.

## 6. Entity selection for implementing blockchain

Building on the preceding analysis, this section aggregates and ranks the aforementioned dual-cost thresholds to delineate distinct threshold-based scenarios, thereby identifying the optimal implementing entity for blockchain adoption within each scenario.

### 6.1. Threshold-based scenario classification

According to Lemmas 1–5 in the previous section, five scenarios are categorized based on the above thresholds which actually reflect the relative scale between the fixed deployment costs and variable operational costs in blockchain implementation. Moreover, the economic willingness of corresponding supply chain entities to implement blockchain in each scenario can be deduced, as listed in Table 4.

**Table 4:** Classified scenarios according to dual-cost thresholds.

Scenarios	Dual-cost thresholds	Economic implications	Economic willingness
I	$v>{\overset{―}{b}}_{\text{1}}\left(f\right)$	Extremely high operational costs	None
II	${\overset{―}{b}}_{\text{2}}\left(f\right)<v<{\overset{―}{b}}_{\text{1}}\left(f\right)$	Moderately high operational costs	Retailer: initiatively
III	${\overset{―}{b}}_{\text{3}}\left(f\right)<v<{\overset{―}{b}}_{\text{2}}\left(f\right)$	Balanced deployment and operational costs	Retailer: initiatively Manufacturer: initiatively
IV	${\overset{―}{b}}_{\text{4}}\left(f\right)<v<{\overset{―}{b}}_{\text{3}}\left(f\right)$	Moderately high deployment costs	Retailer: initiatively, reactively Manufacturer: initiatively
V	$v<{\overset{―}{b}}_{\text{4}}\left(f\right)$	Extremely high deployment costs	Retailer: initiatively, reactively Manufacturer: initiatively, reactively

The following subsections investigate who should be selected to implement blockchain in each scenario. Table 4 only lists the economic willingness in each scenario, which differs from actual extrinsic behaviors. When both the retailer and the manufacturer favor initiative willingness, the equilibrium outcome is that either party is chosen at random to implement blockchain independently. If both the retailer and the manufacturer favor reactive willingness, the behavioral outcome will necessarily that both implement blockchain jointly. If the retailer prefers initiative willingness while the manufacturer favors reactive willingness, or if the manufacturer favors initiative willingness while the retailer prefers reactive willingness, both of which are inherently unstable, the behavioral outcome will inevitably that no entity implements whatsoever. Thus, the interaction between economic willingness and extrinsic behaviors determines the selection of the implementing entity.

### 6.2. Scenario of extremely high operational costs

In this scenario, the blockchain implementation costs fall within the region defined by the dual-cost threshold $\Omega_{\text{1}}=\left\{\left(v,f\right)|{\overset{―}{b}}_{\text{1}}\left(f\right)<v\right\}$. The operational costs, when compared with the deployment costs, are so high that they exceed the first ceiling threshold in Lemma 1. Then, no entity is willing to implement blockchain initiatively or reactively according to Lemmas 1 and 5.

**Proposition 3.** *In the scenario* $\Omega_{\text{1}}=\left\{\left(v,f\right)|{\overset{―}{b}}_{\text{1}}\left(f\right)<v\right\}$ *where the operational costs of implementing blockchain are extremely high, the best choice for green agricultural supply chains is not to implement blockchain.*

If the operational costs are excessively high, neither the manufacturer nor the retailer is inclined to implement blockchain. In fact, during the early phase of blockchain technology development, restricted industrial scale and high incremental costs make it common for supply chain enterprises not implement blockchain spontaneously.

### 6.3. Scenario of moderately high operational costs

In this scenario, the blockchain implementation costs fall within the region defined by the dual-cost threshold $\Omega_{\text{2}}=\left\{\left(v,f\right)|{\overset{―}{b}}_{\text{2}}\left(f\right)<v<{\overset{―}{b}}_{\text{1}}\left(f\right)\right\}$. The operational costs, when compared with the deployment costs, are moderately high, that is, between the first ceiling threshold in Lemma 1 and the second one defined in Lemma 2. Only the retailer is willing to implement blockchain initiatively according to Lemmas 1, 2, and 5.

**Proposition 4.** *In the scenario* $\Omega_{\text{2}}=\left\{\left(v,f\right)|{\overset{―}{b}}_{\text{2}}\left(f\right)<v<{\overset{―}{b}}_{\text{1}}\left(f\right)\right\}$ *where the operational costs of implementing blockchain are moderately high, the best choice for green agricultural supply chains is to select the retailer to implement blockchain independently.*

Furthermore, it also represents the only viable option. Although required to cover blockchain implementation costs, the retailer can secure considerable economic returns. On one hand, implementing blockchain can expand market demand. Specifically, blockchain technology alleviates consumer distrust toward product green attributes, thereby boosting market appetite. On the other hand, implementing blockchain yields potential reputational gains. Blockchain technology is useful for elevating corporate reputation and consolidate consumer loyalty, facilitating sustainable business growth and long-term payoffs. For the retailer, when blockchain operational costs remain at a moderate level, the combined gains from the above two channels may outweigh implementation expenditures. Under such circumstances, the retailer is willing to implement blockchain independently while manufacturers are not.

### 6.4. Scenario of balanced deployment and operational costs

In this scenario, the blockchain implementation costs fall within the region defined by the dual-cost threshold $\Omega_{\text{3}}=\left\{\left(v,f\right)|{\overset{―}{b}}_{\text{3}}\left(f\right)<v<{\overset{―}{b}}_{\text{2}}\left(f\right)\right\}$. The deployment costs balance with operational costs, just between the second ceiling threshold in Lemma 2 and the third one in Lemma 3. Either the manufacturer or the retailer is willing to implement blockchain initiatively according to Lemmas 2, 3, and 5.

**Proposition 5.** *In the scenario* $\Omega_{\text{3}}=\left\{\left(v,f\right)|{\overset{―}{b}}_{\text{3}}\left(f\right)<v<{\overset{―}{b}}_{\text{2}}\left(f\right)\right\}$ *where the deployment costs of implementing blockchain balance with the operational costs, the best choice for green agricultural supply chains is to randomly select one between the manufacturer and the retailer to implement blockchain independently.*

Although both parties are willing to implement blockchain initiatively even if their partner takes no action, neither the manufacturer nor the retailer is willing to implement blockchain reactively after their counterpart has already done so. When the manufacturer remains uninvolved, the retailer intends to launch blockchain deployment on its own because the combined gains from expanded market revenue and reputational benefits as a first mover exceed the associated implementation expenses. Nevertheless, once the manufacturer moves first, the retailer is reluctant to follow reactively since the marginal revenue and reputational gains obtained as a follower cannot offset the implementation costs. The same logic applies to the manufacturer. Thus, it is evident that the economic willingness of the manufacturer and the retailer to implement blockchain is mutually exclusive. Accordingly, the green agricultural supply chain can only select a single entity to implement blockchain independently, with the final choice being stochastic. The ultimate participant hinges on their timing rivalry. The entity more responsive to market shifts and technological evolution is more likely to secure first-mover advantage in implementing blockchain initiatively.

### 6.5. Scenario of moderately high deployment costs

In this scenario, the blockchain implementation costs fall within the region defined by the dual-cost threshold $\Omega_{\text{4}}=\left\{\left(v,f\right)|{\overset{―}{b}}_{\text{4}}\left(f\right)<v<{\overset{―}{b}}_{\text{3}}\left(f\right)\right\}$. The deployment costs, when compared with the operational costs, are moderately high, that is, between the third ceiling threshold in Lemma 3 and the fourth one in Lemma 4. According to Lemmas 3, 4, and 5, not only the manufacturer but also the retailer is willing to implement blockchain initiatively, and the retailer is willing to implement it reactively while the manufacturer is not willing to implement it reactively.

**Proposition 6.** *In the scenario* $\Omega_{\text{4}}=\left\{\left(v,f\right)|{\overset{―}{b}}_{\text{4}}\left(f\right)<v<{\overset{―}{b}}_{\text{3}}\left(f\right)\right\}$ *where the deployment costs of implementing blockchain are moderately high, the best choice for green agricultural supply chains is to select the retailer to implement blockchain independently.*

Although there are theoretically three possibilities, the retailer’s initiative implementation, the retailer’s reactive implementation, and the manufacturer’s initiative implementation, only the retailer’s initiative implementation can achieve a stable equilibrium. First, the state where the retailer implements blockchain independently is stable. If the retailer opts to implement initiatively, the manufacturer will not follow suit due to the lack of reactive willingness to do so. Since the retailer is indeed willing to implement even without the manufacturer’s participation, the final equilibrium outcome is that the retailer implements blockchain solely. Second, the state where the manufacturer implements blockchain independently is unstable. Once the manufacturer has implemented, the retailer endowed with reactive willingness inevitably follows up with implementation, thereby disrupting the equilibrium. Third, the state where the retailer implements blockchain reactively is infeasible. If the retailer follows up after the manufacturer has implemented, the manufacturer will withdraw because the manufacturer is willing to do so independently but is not willing to do so jointly. In this case, the retailer will replace the manufacturer to implement blockchain initiatively rather than reactively. Therefore, by synthesizing the stability and practicability of above three aspects, the green agricultural supply chain should select the retailer instead of the manufacturer to implement blockchain independently.

### 6.6. Scenario of extremely high deployment costs

In this scenario, the blockchain implementation costs fall within the region defined by the dual-cost threshold $\Omega_{\text{5}}=\left\{\left(v,f\right)|0<v<{\overset{―}{b}}_{\text{4}}\left(f\right)\right\}$. The deployment costs, when compared with the operational costs, are extremely high, even more than the fourth ceiling threshold in Lemma 4. According to Lemmas 4 and 5, not only the manufacturer but also the retailer is willing to implement blockchain either initiatively or reactively.

**Proposition 7.** *In the scenario* $\Omega_{\text{5}}=\left\{\left(v,f\right)|0<v<{\overset{―}{b}}_{\text{4}}\left(f\right)\right\}$ *where the deployment costs of implementing blockchain are extremely high, the best choice for green agricultural supply chains is to select both the manufacturer and the retailer to implement blockchain jointly.*

Similarly, although there are theoretically three possibilities, the retailer’s independent implementation, the manufacturer’s independent implementation, and joint implementation by both the manufacturer and the retailer, only the joint implementation can achieve a stable equilibrium, whereas the states in which either the manufacturer or the retailer implements blockchain independently are unstable. For instance, when the manufacturer has implemented blockchain, the retailer will follow suit due to its reactive willingness to do so. The manufacturer will also continue implementing because it is likewise willing to act reactively. Thus, the final equilibrium outcome is that both parties implement blockchain jointly. When the retailer has implemented blockchain, the manufacturer will also follow up, and the two will ultimately end up with joint implementation. Consequently, the green agricultural supply chain should select both the manufacturer and the retailer to implement it jointly.

### 6.7. Consolidation of all scenarios

By aggregating the above five scenarios, the best entity choice for implementing blockchain in each scenario is listed in Table 5 respectively. Generally, when both fixed deployment costs and variable operational costs are excessively high, no supply chain entity is willing to implement blockchain technology. Similarly, an excessively high level of either fixed costs or variable costs alone also discourages blockchain adoption. By contrast, blockchain implementation becomes economically feasible only under two cost-matching configurations: relatively high fixed costs matched with relatively low variable costs, or relatively low fixed costs matched with relatively high variable costs. Only such cost configurations can motivate supply chain entities to implement blockchain. More specifically, for the extremely high operational costs, referring to scenario $\Omega_{\text{1}}$, the best choice is to reject implementing blockchain. For the extremely high deployment costs, referring to scenario $\Omega_{\text{5}}$, the best choice is to select both the manufacturer and the retailer to implement it jointly. In other scenarios, the retailer ($\Omega_{\text{2}}$, $\Omega_{\text{3}}$, and $\Omega_{\text{4}}$), or occasionally the manufacturer ($\Omega_{\text{3}}$), should be selected to implement blockchain independently.

**Table 5:** Entity selection for implementing blockchain in each scenario.

Scenarios	Definitions	Best entity choice	Implementation ways
$\Omega_{\text{1}}$	Extremely high operational costs	None: select nobody	rejectingly
$\Omega_{\text{2}}$	Moderately high operational costs	R: select the retailer	independently
$\Omega_{\text{3}}$	Balanced deployment and operational costs	M or R: randomly select the manufacturer or the retailer	
$\Omega_{\text{4}}$	Moderately high deployment costs	R: select the retailer	
$\Omega_{\text{5}}$	Extremely high deployment costs	M and R: select both the manufacturer and the retailer	jointly

By comparing Tables 4 and 5, it can be seen that the entity selection is determined by the interaction of the manufacturer’s economic willingness and the retailer’s.

In scenario $\Omega_{\text{1}}$, the operational costs of implementing blockchain are excessively high. It is infeasible for reputational gains to offset both deployment and operational costs. Neither the manufacturer nor the retailer can attain positive profits when implementing blockchain, and thus neither has any economic motivation to do so. Therefore, the agricultural supply chain should abstain from implementing blockchain technology, and no entity should be selected.

In scenario $\Omega_{\text{2}}$, the operational costs of implementing blockchain are moderately high. For the retailer, it is viable to offset both deployment and operational costs even if the manufacturer refuses to implement blockchain, and the retailer has to do so independently. The retailer will definitely possess initiative economic willingness. For the manufacturer, it is impossible to offset both operational and deployment costs even if the retailer agrees to implement blockchain jointly. The manufacturer will neither implement it independently nor jointly with the retailer, and thus exhibits neither initiative nor reactive economic willingness. Therefore, the retailer should be selected to implement blockchain independently because only the retailer displays initiative willingness.

In scenario $\Omega_{\text{3}}$, the operational costs and deployment costs of implementing blockchain are relatively balanced. For the retailer, it is feasible to offset both deployment and operational costs even if the manufacturer refuses to implement blockchain. However, once the manufacturer agrees to implement it jointly, the retailer will decline. Thus, the retailer is willing to act independently rather than jointly. For the manufacturer, the situation is identical. As a result, although both parties demonstrate initiative willingness, only a unilateral initiative constitutes an equilibrium. Thus, either the manufacturer or the retailer should be randomly selected to implement blockchain independently.

In scenario $\Omega_{\text{4}}$, the deployment costs of implementing blockchain are moderately high. For the retailer, it is feasible to offset both deployment and operational costs even if the manufacturer declines to adopt blockchain. Furthermore, should the manufacturer agree to joint implementation, the retailer will accede accordingly. Consequently, the retailer is willing to act either independently or collaboratively. For the manufacturer, however, it is impossible to offset both operational and deployment costs, even if the retailer consents to joint implementation. The manufacturer will opt for neither independent nor joint implementation, and thus demonstrates neither initiative nor reactive economic willingness. Although the retailer exhibits both proactive and reactive willingness, while the manufacturer only shows initiative willingness, only the retailer’s unilateral initiative constitutes an equilibrium. Thus, the retailer should be selected to implement blockchain independently.

In scenario $\Omega_{\text{5}}$, the deployment costs of implementing blockchain are excessively high. For the retailer, it is feasible to offset both deployment and operational costs even if the manufacturer declines to adopt blockchain. However, should the manufacturer agree to joint implementation, the retailer will opt out. The situation is analogous for the manufacturer. Consequently, although both the manufacturer and the retailer exhibit both initiative and reactive willingness simultaneously, only their bilateral homogeneous willingness constitutes an equilibrium. Thus, both should be selected to implement it collaboratively.

Additionally, it should be emphasized that although the specific values and regions of the scenarios classified and enclosed by the dual-cost thresholds vary with the demand parameters including α and β, the ranking of optimal implementers remains unchanged. In other words, the optimal implementer pertaining to each scenario stays the same, while only the occurrence probability of each scenario undergoes slight adjustments. Derived and rigorously proven via mathematical deduction under the model assumptions, the fundamental properties of the aforementioned optimal implementers in the corresponding scenarios are invariant to changes in the value of any single parameter. Of course, the parameters including but not limited to α and β must be constrained by conventional feasible boundary conditions, which are required by positive product prices, output quantities, and equilibrium profits of all supply chain participants.

## 7. Visualized verification

This section presents the visually illustrated economic willingness to implement blockchain and the best entity selection for implementing blockchain in the above five scenarios classified by dual-cost thresholds. The above theoretical thresholds derived from rigorous mathematical reasoning under corresponding assumptions are universally applicable, while the following case study and the observations from it only provide a specific industrial instantiation of such conclusions.

### 7.1. Case study

Guizhou Province, located in the southwest of China, is famous as the home to pepper, and is endowed with China’s largest hot pepper industry, a cornerstone of the regional agricultural economy. With a stable planting area of 5 million acres and an annual output of 7 million tons, the hot pepper industry has generated 28 billion yuan in primary output and 18 billion yuan in the relevant pepper processing industry. As green development becomes a top priority, leading manufacturers adopt blockchain technology as a core enabler for more environmentally-friendly agricultural practices.

ZH Food, a key participant in Guizhou’s hot pepper supply chain, has taken the lead in integrating premium seeds, advanced technologies, and green cultivation methods throughout the entire process to improve the environmental performance of pepper production. The company focuses on renowned local high-yield varieties, achieving an intensive seedling survival rate of over 90% on large-scale standardized bases and ensuring 100% coverage of top-tier cultivars. To promote green production, ZH Food advocates innovative techniques such as floating seedling raising, mechanical ridging and mulching, mechanized direct seeding and transplanting, and precision irrigation and fertilization. To ensure the credibility and traceability of green hot pepper production, ZH Food has built a comprehensive blockchain system. Critical data spanning land use, seed breeding, pesticide and fertilizer application, harvesting, and logistics are all uploaded to the blockchain, creating an unchangeable record of the entire process. These initiatives have led to the establishment of more than 100 ecological cultivation demonstration sites by ZH Food, significantly boosting both the sustainability and market trustworthiness of its hot pepper products. A prime example is the ZUNJIAO 222 variety, which achieves a doubled yield of 1500 kg per acre compared to the standard 750 kg, while complying with strict environmental standards.

As a top-tier producer in the hot pepper supply chain, ZH Food supplies Guizhou hot peppers nationwide and globally through offline and online channels. Key distribution platforms include the China Hot Pepper City, the country’s biggest hot pepper trading hub, and MaiLa.com, a specialized e-commerce platform that incorporates blockchain technology. These platforms not only boost commodity circulation but also leverage blockchain to promote and verify the authenticity of high-quality green hot peppers.

### 7.2. Visualizations

A numerical example is designed to visually illustrate the above theoretical findings. According to the standardized data of the above hot pepper supply chain, that is, the agricultural supply chains discussed in [45], let potential demand a=500, price sensitivity α=0.6, unit production c=30, marginal coefficient of green investment k=50, green sensitivity β=0.8, deployment costs within the interval 200≤f≤400. Furthermore, according to the empirical measurement by [46], let ξ=0.1 and g=0.01.

**Fig 2:**
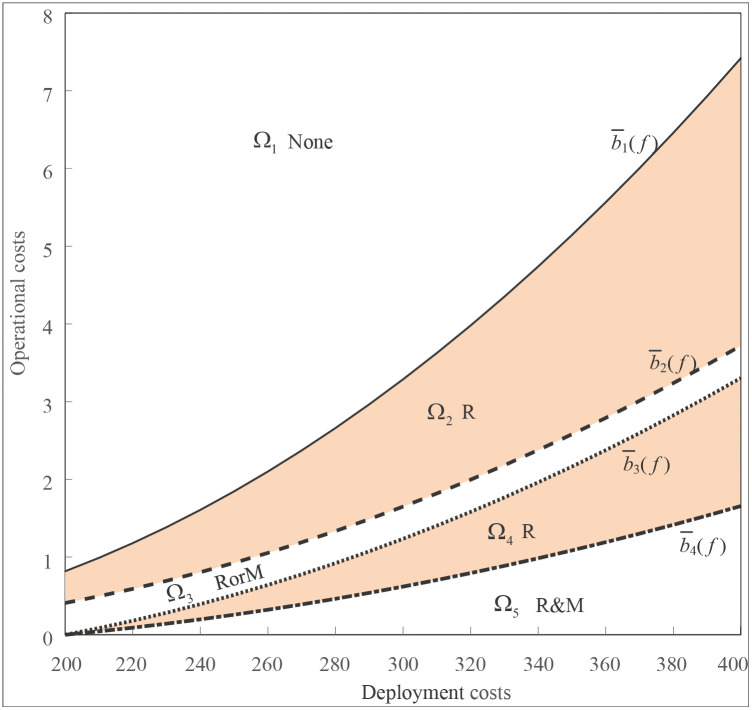
Visualized scenarios with selected entities.

Then, the regions corresponding to each scenario are illustrated in Fig 2 by means of a coordinate system with operational costs and deployment costs as the X-axis and Y-axis, respectively. Curve ${\overset{―}{b}}_{i}\left(f\right)\text{(}i=\text{1, 2, 3, 4)}$ denotes the ceiling thresholds defined in Lemmas 1–4. Region $\Omega_{i}\text{(}i=\text{1, 2, 3, 4,5)}$ denotes the scenarios sorted by the dual cost criteria, and these scenarios have been classified and presented in Tables 3 and 4.

Fig 2 shows the influence of operational costs and deployment costs on the implementer selection of blockchain in the agricultural supply chain with standardized data. The optimal blockchain implementer varies with different combinations of operational costs and deployment costs. The suitable implementer for blockchain adoption in different regions enclosed by the two types of costs is visually presented.

It is both meaningful and important to clarify how the operational and deployment costs are evaluated in practice. Rather than comparing their absolute values directly, we compare the operational costs to a certain proportion of the deployment costs because the deployment and operational costs typically differ by orders of magnitude. Specifically, a certain percentage of deployment costs is set as the benchmark to assess whether operational costs are relatively high or low. For example, in the above case study, when the deployment costs of implementing blockchain equal 300, the benchmark should be set at only 1% of the deployment costs in absolute terms according to Proposition 3. Thus, the operational costs would be judged as extremely high only if they reach 3. Such a method is popular in practical and academic paradigms [43].

In region $\Omega_{\text{1}}$ enclosed by Curve ${\overset{―}{b}}_{\text{1}}\left(f\right)$ and the upper limit, the operational costs of implementing blockchain, compared with the deployment costs, are extremely high, and thus no entity is willing to implement it. The best entity selection is denoted as None.

In region $\Omega_{\text{2}}$ bounded by Curve ${\overset{―}{b}}_{\text{2}}\left(f\right)$ and ${\overset{―}{b}}_{\text{1}}\left(f\right)$, the operational costs of implementing blockchain are moderately high, only the retailer is willing to do so, and thus the retailer should be selected to implement blockchain independently. The best entity selection is designated as R.

In region $\Omega_{\text{3}}$ bounded by Curve ${\overset{―}{b}}_{\text{3}}\left(f\right)$ and ${\overset{―}{b}}_{\text{2}}\left(f\right)$, the operational costs of implementing blockchain align with the deployment costs at a relative balance, and thus either the manufacturer or the retailer is willing to implement blockchain initiatively. The manufacturer and the retailer should be selected randomly to do so independently. The best entity selection is denoted as R or M.

In region $\Omega_{\text{4}}$ bounded by Curve ${\overset{―}{b}}_{\text{4}}\left(f\right)$ and ${\overset{―}{b}}_{\text{3}}\left(f\right)$, the deployment costs of implementing blockchain, compared with the operational costs, are moderately high. Although the retailer is willing to do so either initiatively or reactively while the manufacturer is willing to do so initiatively rather than reactively, only the retailer’s independent implementation can reach a stable equilibrium. Thus, the retailer should be selected to implement blockchain independently. The best entity selection is denoted as R.

In region $\Omega_{\text{5}}$ bounded by the lower bound and Curve ${\overset{―}{b}}_{\text{4}}\left(f\right)$, the deployment costs of implementing blockchain, compared with the operational costs, are too high. Although not only the manufacturer but also the retailer is willing to implement it either initiatively or reactively, only the joint implementation is stable. Thus, both the manufacturer and the retailer should be selected to implement it jointly. The best entity selection is designated as R & M.

To summarize, regions $\Omega_{i}\text{(}i=\text{1, 2, 3, 4,5)}$ visually illustrate and verify the theoretical findings in Propositions 3–7. They further extend these results by showing the relative sizes of the regions, leading to the following Observations.

### 7.3. Observations

Furthermore, the following observations can be drawn based on Fig 2.

**Observation 1.** *The likelihood of not implementing blockchain exceeds 50%.*

Comparing the above five regions, it is clear that the area of region $\Omega_{\text{1}}$ is the biggest, accounting for nearly half of the total area. Therefore, in the examined case study, the green agricultural supply chain actually should, in most instances, refuse to implement blockchain. This is consistent with the practical fact that only a few leading agricultural enterprises implement blockchain, while ordinary supply chain enterprises do not take the initiative to implement blockchain. The reason is that the current scale of the blockchain industry is not large, and the relevant costs are not low enough. Surprisingly but interestingly, this reveals the defect in the traditional assumption in the existing literature that some supply chain entities must implement blockchain.

**Observation 2.** *The potential entity selections for implementing blockchain vary contextually.*

In region $\Omega_{\text{1}}$, no entities should be selected. In region $\Omega_{\text{2}}$ and $\Omega_{\text{4}}$, the retailer is the only entity should be selected. In region $\Omega_{\text{3}}$, the manufacturer and the retailer, as entities implementing blockchain, are alternative choices. In region $\Omega_{\text{5}}$, the manufacturer and the retailer are bound together as implementing entities. It is clear that the area of $\Omega_{\text{1}}$, is remarkably larger than the areas of other regions. Hence, it is most likely that no entity implements blockchain at all, which aligns with the reality that green agricultural supply chains have just begun implementing blockchain, yet blockchain technology itself has not been extensively adopted. This validates and displays Propositions 3–7.

**Observation 3.** *The thresholds of operational costs always exhibit a consistent upward trend as deployment costs increase in each situation.*

Apparently, all ${\overset{―}{b}}_{i}\left(f\right)\text{(}i=\text{1, 2, 3, 4)}$ are strictly rising functions. As the deployment costs increase, the blockchain covers a broader scope and can result in more reputational gains stemming from potential long-term market orders and sales. This may offset relatively higher operational costs, and thus raise the thresholds. Therefore, as supply chain enterprises expand their investment in blockchain, they are willing to tolerate growing operational costs. Notably, it is inconsistent with the widely accepted straightforward understanding of mutually exclusive growth and decline between deployment costs and operational costs. This extends the findings of Propositions 3–7. Additionally, as the thresholds of operational costs always increase with the deployment costs, there is a potential problem of excessive investment where a high deployment cost results in an unsustainable operational cost. This problem is prevented by the restriction on the deployment costs in Assumption 7.

**Observation 4.** *Within dual-cost thresholds, it is most likely that the retailer implements blockchain independently, while it is least likely for the manufacturer to do independently.*

In Regions $\Omega_{\text{2}}$ and $\Omega_{\text{4}}$, the retailer implements blockchain independently. In region $\Omega_{\text{3}}$, either the manufacturer or the retailer implements blockchain independently. In region $\Omega_{\text{5}}$, they implement it jointly. It is clear that the economic willingness of the manufacturer to implement blockchain is significantly weak. One possible reason is that the manufacturer has to undertake investments in green technology to satisfy the consumers’ green preferences. When implementing blockchain independently, the manufacturer needs to bear investment costs in both green technology and blockchain technology alone. Unless the reputational gains happen to offset the operational costs well, the manufacturer is unwilling to implement blockchain initiatively. On the contrary, it is prevalent that the retailer implements blockchain independently. Amazingly and notably, this provides an explanation for the real-world industrial practice where blockchain implementation in most green agricultural supply chains is proactively initiated by retailers (such as JD, Tmall, and Walmart) rather than manufacturers.

Summarily, those Observations synthesize and expand the findings of Propositions 3–7.

### 7.4. Extensions

This subsection examines the sensitivity of optimal entity selection in every scenario to the key parameters, the information vagueness and blockchain-based reputation, respectively.

(1) The impact of information vagueness.

**Fig 3:**
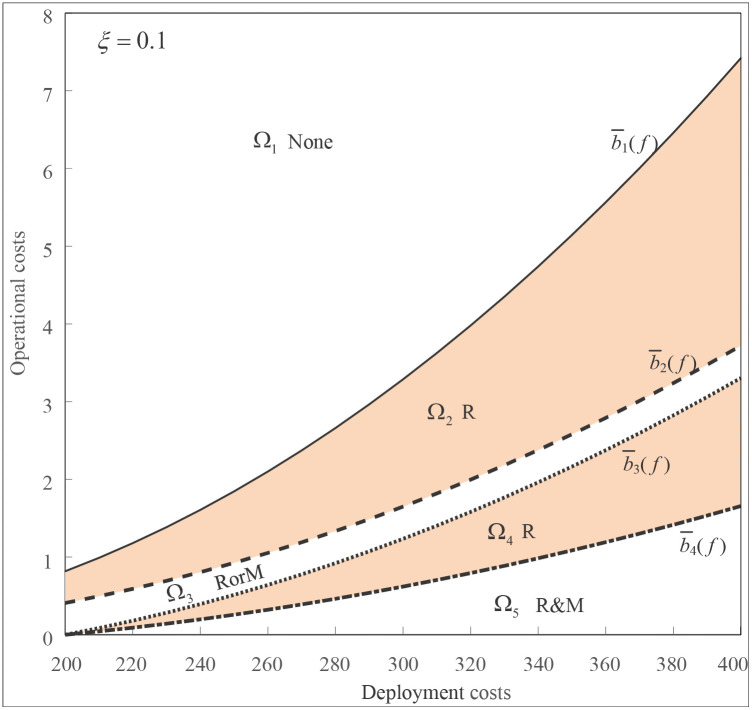
Regions with selected entities whenξ=0.1 holding all other parameters constant.

**Fig 4:**
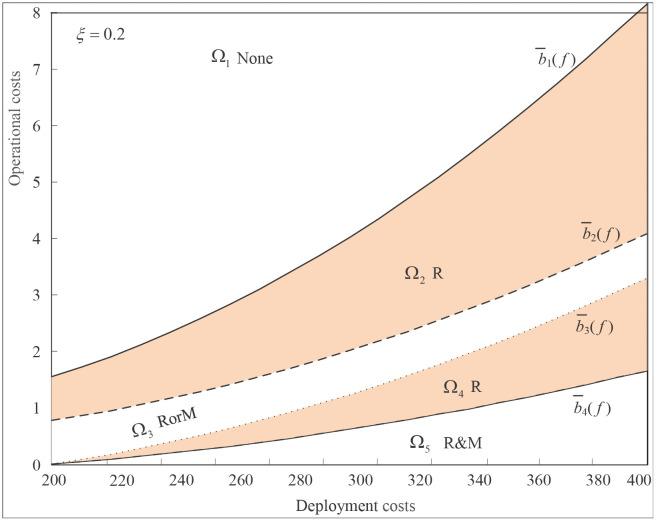
Regions with selected entities when ξ=0.2 holding all other parameters constant.

To illustrate how the vagueness of green information, denoted as ξ, influences the above Regions $\Omega_{i}(i=1,2,3,4,5)$, which reflect the economic willingness to implement blockchain and thus guide the related optimal entity selection, we set ξ=0.2 while holding all other parameters unchanged, whereas Fig 2 above presents the case of ξ=0.1. The new Regions corresponding to each scenario then vary as shown in Fig 3 and Fig 4.

How information vagueness alters the appropriate implementer for blockchain adoption under diverse combinations of operational costs and deployment costs can be clearly concluded by comparing Figs 3 and 4, which correspond to ξ=0.1 and ξ=0.2 respectively.

First, the vagueness of green information can raise the probability of implementing blockchain in agricultural supply chains. Region $\Omega_{\text{1}}$ in Fig 3 where ξ=0.1 is narrower than that in Fig 4 where ξ=0.2, so the probability of refusing to implement blockchain is reduced by more vague green information. The basic logic is as follows: the higher the ambiguity there is in green information, the more blockchain implementation alleviates consumers’ distrust, and the more significant the improvement in sales revenue; the greater the vagueness of green information in the agricultural product market, the more imperative it is to implement blockchain in agricultural supply chains.

Second, the probability of blockchain implementation in each scenario is elevated to various degrees. The probability in selecting the retailer to implement alone is enhanced to the greatest extent, which is manifested by a significant expansion in $\Omega_{\text{2}}$ and an unchanged $\Omega_{\text{4}}$. The probability of randomly selecting the retailer or manufacturer to implement independently has also increased to some extent, which is manifested by a significant expansion of $\Omega_{\text{3}}$. However, the probability of selecting the retailer and manufacturer to implement jointly has declined because Regions $\Omega_{\text{4}}$ and $\Omega_{\text{5}}$ remain unchanged while Regions $\Omega_{\text{2}}$ and $\Omega_{\text{3}}$ expand.

In summary, it verifies and expands Corollary 4 by showing how information vagueness affects the corresponding regions which in fact determine the selection of optimal blockchain implementers.

(2) The impact of blockchain technological reputation.

**Fig 5:**
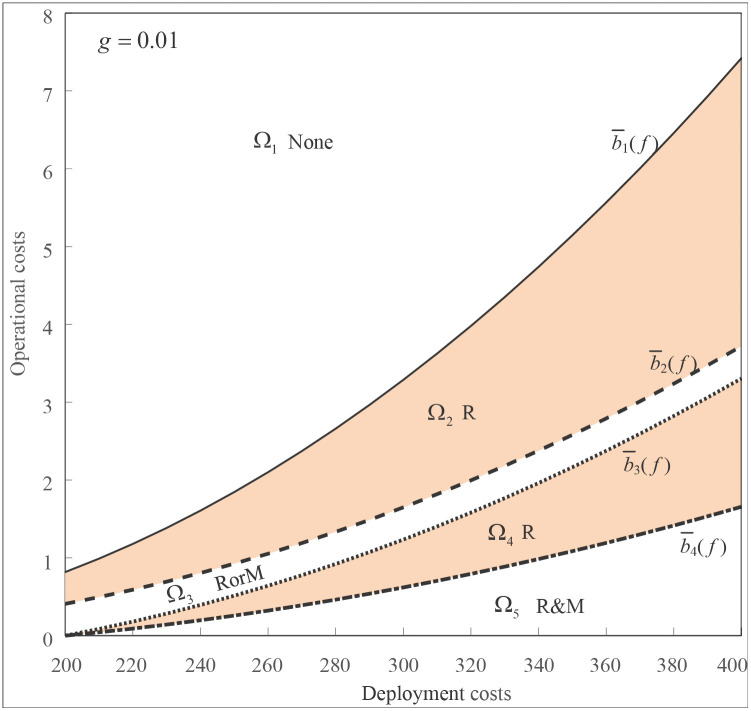
Regions with selected entities when g=0.01 holding all other parameters constant.

**Fig 6:**
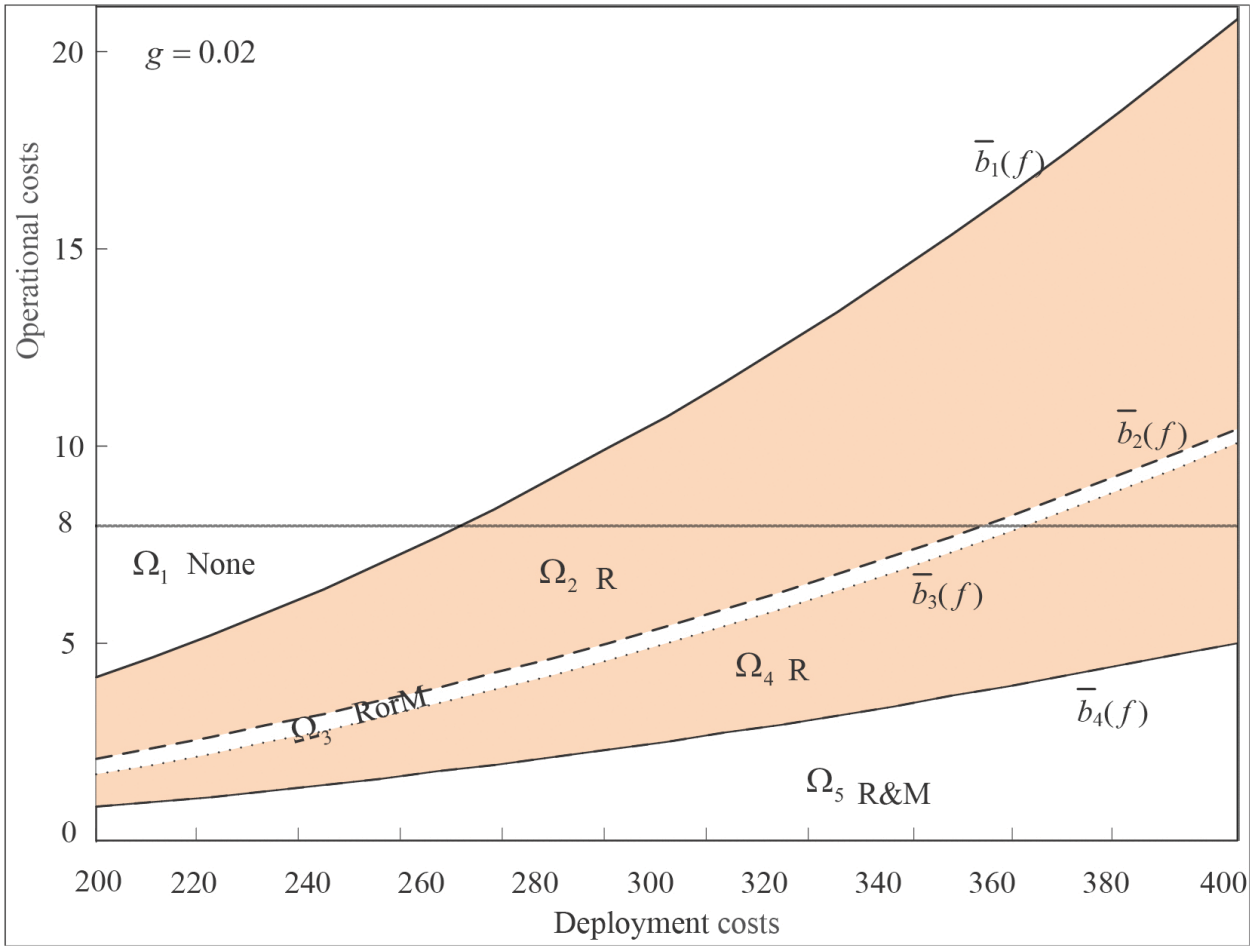
Regions with selected entities when g=0.02 holding all other parameters constant.

To illustrate how the marginal blockchain reputation denoted as g influences the above Regions $\Omega_{i}(i=1,2,3,4,5)$, we set g=0.02 while holding all other parameters unchanged, whereas Fig 2 shows the case of g=0.01. Then, Regions corresponding to each scenario change as shown in Figs 5 and 6, where the line of operational costs equaling 4 is given as a benchmark for comparison with Fig 2.

How blockchain-based technological reputation affects the appropriate implementer for blockchain adoption under the range of combinations of operational costs and deployment costs can be clearly concluded by comparing Figs 5 and 6, which correspond to g=0.01 and g=0.02 respectively.

First, the technological reputation can remarkably expand the applicable fields for blockchain implementation. The feasible area enclosed by operational costs and deployment costs is substantially broadened, which reduces the economic willingness threshold and makes blockchain technology more admissible. A stronger technological reputation can bring indirect and long-term benefits, thereby compensating higher implementation costs.

Second, the probability of blockchain implementation in each scenario is boosted to different degrees. Regions $\Omega_{\text{1}}$ to $\Omega_{\text{5}}$ all grow in size. In particular, even the seemingly narrow and slim $\Omega_{\text{3}}$ also expands noticeably. However, the degrees of improvement vary significantly in each scenario. The probability of selecting the manufacturer to implement blockchain independently remains low, the probability of selecting both the retailer and manufacturer to implement blockchain jointly is slightly raised, while the probability of selecting the retailer to implement blockchain independently is enhanced substantially.

Summarily, it verifies and expands Corollaries 4 and 5 by showing how blockchain technological reputation affects the corresponding regions that actually determine the selection of optimal blockchain implementers.

## 8. Conclusion

### 8.1. Main findings

This paper establishes four game theoretic models featuring distinct blockchain implementation in green agricultural supply chains to investigate the economic willingness to implement blockchain and assess the entity selection under various scenarios. Different from previous literature focusing merely on unilateral implementation strategies, this paper pays attention to the economic rationality of blockchain adoption decisions, addressing the ambiguities in entity selection. The key findings are synthesized as follows.

First, both manufacturers and retailers may contingently exhibit initiative or reactive willingness to implement blockchain in green agricultural supply chains across different scenarios. This lays an important theoretical foundation for understanding heterogeneous blockchain adoption behavior. By initiative willingness, firms still implement blockchain even when their supply chain partners have not yet done so. On the contrary, firms only follow suit after their partners have already adopted the blockchain technology by reactive willingness. Regardless of an independent or joint approach, our results confirm that the downstream retailer is more willing to implement blockchain than the upstream manufacturer, where both manufacturers and retailers are willing to implement blockchain only when costs fall within the dual thresholds. Once beyond the thresholds, no participant in green agricultural supply chains is willing to do so. Notably, at the current stage of blockchain technology development, in which the industrial scale is small and implementation costs are relatively high, supply chain enterprises rarely implement blockchain spontaneously in most practical instances. This empirical evidence effectively explains the low penetration rate of blockchain technology in current green agricultural supply chains and further highlights the limitations of existing research. The conventional paradigm in previous literature always assumes that there must be some mandatorily designated entities implementing blockchain but ignores their economic intention. Our findings demonstrate that such a simplistic assumption is theoretically unreasonable and practically inapplicable, which remedies the research bias of ignoring economic intentions in existing blockchain adoption literature and provides a more realistic analytical paradigm for subsequent related studies.

Second, this study further clarifies that the selection of blockchain implementation entities is contextually determined by deployment and operational dual-costs. If the operational costs are extremely high, no entity should be selected. If the deployment costs are extremely high, both manufacturers and retailers should be selected to implement blockchain jointly. Otherwise, when both costs are moderate, retailers should be selected to implement blockchain independently. In particular, manufacturers can also independently implement blockchain if deployment costs are well matched with operational costs. A key contribution of this research is the construction of dual-cost thresholds defined by the fixed deployment and variable operational costs within a two-dimensional coordinate system. But previous literature usually examines only one dimension, either the fixed or the variable costs of implementing blockchain, which overlooks the interactive effect of dual costs and cannot fully reflect real-world decision-making logic. In this regard, such two-dimensional dual-cost threshold framework innovatively integrates coupled cost factors, fills the research gap of single-dimensional cost analysis in blockchain literature, and significantly improves the explanatory power of enterprise blockchain implementation behaviors in green supply chains across various scenarios.

Third, the feasibility of different entity selection strategies varies substantially within the dual-cost thresholds. The most likely scenario is retailers’ independent implementation, followed by their joint implementation, and the least likely is manufacturers’ independent implementation. This provides a reasonable theoretical explanation for real-world industrial practice, whereby major retail platforms such as JD, Tmall, and Walmart proactively implement blockchain. Whether acting as manufacturers or retailers implementing blockchain, both parties favor independent implementation rather than joint efforts. Importantly, this outcome suggests that the widely held intuitive view that blockchain should be implemented through cooperation maybe actually economically irrational. This subverts the stereotyped cognition of cooperative implementation being superior in traditional supply chain digital governance research and hence provides practical guidance for green agricultural supply chain enterprises to formulate rational blockchain technology strategies. Furthermore, once beyond the dual-cost thresholds, it is impossible for any entity to implement blockchain in green agricultural supply chains. The case study further verifies the validity of the threshold model, showing that the probability of infeasible implementation exceeds 50%, which aligns well with the current industry reality that only a small number of supply chain enterprises have formally adopted blockchain technology. Such verification further proves the robustness and practical applicability of our research conclusions, making the findings more referential for industrial practice and policy formulation.

Additionally, it is important to emphasize that the above findings are contingent on the underlying model assumptions. For instance, the reputational gains for implementing blockchain are based on the premise that the current development and application of blockchain technology are still in the initial high-growth stage of the classic three-stage model. China issued the *Guiding Opinions on Accelerating the Application of Blockchain Technology and Industrial Development* in 2021 and the *Plan for Accelerating the Building of a Strong Agricultural Nation* in 2025. The application of blockchain technology in green agricultural supply chains is currently developing rapidly, which aligns well with the characteristics of this initial stage. In a few coming years, as blockchain technology achieves wider implementation and popularization, the industry will transition into the second stage. At that point, the configuration and conclusions of this study should be revised accordingly. Moreover, the assumption of perfect transparency of product greenness upon blockchain adoption does not limit the generalizability of the above findings because the perfect transparency assumption is essentially equivalent to the partial transparency scenario, as elaborated in the subsection for Assumption 5. We employ the perfect transparency assumption solely for mathematical simplicity, with no impact on the generalizability of the results. Overall, this study systematically reveals the cost-dependent heterogeneous implementation rules of blockchain in green agricultural supply chains, provides a rigorous theoretical basis and practical decision-making reference for promoting the sustainable and high-quality application of blockchain technology in green agriculture, and offers valuable insights for subsequent interdisciplinary research on green supply chain digital transformation.

The core conclusions are fundamentally obtained through rigorous theoretical deduction built on the game theory model, and the research outcomes must be explained and applied strictly within the scope of the model’s initial assumptions and reductions. Although they are universally applicable under corresponding assumptions, practical cases are required for concrete demonstration. The case study of Guizhou pepper supply chain only serves as a single empirical verification example, which cannot provide sufficient comprehensive practical evidence to universally validate the model outputs across diverse agricultural supply chains. For this reason, the findings inevitably carry boundary constraints tied to preset model assumptions. Thus, richer multi-region, multi-crop empirical data should be collected in follow-up studies to enrich practical verification.

### 8.2. Practical implications

Some practical implications can be drawn for policymakers of agricultural blockchain technology, practitioners of green agricultural supply chains, and consumers of green agricultural products, respectively.

First, the practical implications for policymakers regarding agricultural blockchain technology encompass three sub-aspects. Firstly, construct and refine public cross-departmental data infrastructure. The improvement of public infrastructure can generate external economies, and thereby lowering the deployment costs for entities to implement blockchain in agricultural supply chains. This expands the Region Ω_5_ and reduces Ω_1_, broadening the scope within which specific entities are willing to implement blockchain. Secondly, subside leading agricultural retailing enterprises to implement blockchain actively. Sales volume-based subsidies can reduce the operational costs of blockchain system, which shrinks the Region Ω_1_ and Ω_2_. In return, this encourages large agribusinesses to adopt blockchain technology, promote the standardized storage and transmission of data, expand the connected scope, and foster economies of scale. Thirdly, advocate the concept of green consumption of agricultural products. Although non-economic and indirect, green consumption awareness can both promote the green planting of agricultural products and drive demand for certification and traceability of green information. Ultimately, this enhances the market-driven dual motivation for blockchain implementation.

Second, the practical implications for practitioners in green agricultural supply chains can be divided into three subcategories. Firstly, timely agricultural product data should be collected and systematically organized. Crops are characterized by long production cycles, long transportation distances, and diverse types of relevant information. The collection, sorting, transformation and coding of various information throughout the whole lifecycle facilitates the traceability, monitoring and sharing of product information through blockchain technology. Secondly, capture market dynamics and seize opportunities for blockchain adoption. In cases where product information is severely distorted during transmission, consumers harbor strong distrust toward product green attributes, and there is an urgent market demand for transparency and traceability regarding origin, quality certification and other attributes, blockchain technology should be adopted in a timely manner. Thirdly, comprehensively balance the dual costs and benefits of blockchain implementation. Based on the aforementioned scenario classification, benchmark criteria for cost evaluation should be reasonably formulated according to local industrial conditions. Blockchain should only be implemented when operational costs are below the predefined proportion of deployment costs. Direct benefits originate from market expansion driven by reduced information distortion, while indirect potential benefits arise from gradually accumulated intangible reputation and competitive advantages. Overall, costs and benefits should be carefully weighed and balanced.

Third, the practical implications for consumers of green agricultural products consist of three sub-elements. Firstly, raise demands for the traceability of product information. High standards of transparency urge blockchain-based platforms to make real-time data on crop origin, processing steps, and transportation accessible to consumers, which mitigates information asymmetry and thereby cultivates solid consumer trust. Secondly, encourage active engagement in quality feedback mechanisms. Via a blockchain-based system, consumers can submit reviews or report issues directly within the supply chain network, which spurs manufacturers to improve product quality proactively. Thirdly, seek all-round protection of consumer rights through immutable transaction records. In instances of product quality disputes, blockchain-stored data can provide consumers with authoritative evidence, while smart contracts can automatically trigger compensation processes to enhance responsibility supervision.

### 8.3. Future research directions

There are some limitations, such as the lack of consideration for power structures and non-economic factors in green agricultural supply chains. The decision of whether to implement blockchain is a comprehensive outcome derived from balancing economic benefits and diverse non-economic factors, which mainly include governmental policies, organizational dynamics, regulatory frameworks and social preferences. How these factors collectively shape the final implementation decision of blockchain technology will be explored in future research. First, incorporate various power structures and compare the entity selections in manufacturer-led and retailer-led green agricultural supply chains. Second, incorporate non-economic factors, such as governmental policies, organizational dynamics, regulatory frameworks and social preferences, and explore how they affect the entity selection for implementing blockchain. Third, incorporate the competition and multi stages in agricultural supply chains, and investigate how they change the economic willingness of implementing blockchain.

## Supporting information

S1 Appendix Proof of Theorems and Lemmas. (DOCX)
