# Supplementary material for: Selecting suitable entities to implement blockchain in green agricultural supply chains
Source: PLoS One. 2026 Jul 6;21(7):e0334867. doi: 10.1371/journal.pone.0334867 (PMC13336196; doi:10.1371/journal.pone.0334867)
Supplement: S1 Appendix — (DOCX) [file pone.0334867.s001.docx]

Supporting information

Appendix S

Appendix S.1: Proof of Theorem 1

Substituting $D_{N}=a-\alpha p+\beta(1-\xi)e$ into $\pi_{m}^{A}=(w-c)D_{N}-\frac{1}{2}ke^{2}$ and $\pi_{r}^{A}=(p-w)D_{N}$, we yield $\pi_{m}^{A}=(w-c)(a-\alpha p+\beta e(1-\xi))-\frac{1}{2}ke^{2}$ and $\pi_{r}^{A}=(p-w)(a+\beta e(1-\xi)-\alpha p)$. The first derivatives and the second derivatives of $\pi_{r}^{A}$ with respect to $p$ are obtained as $\frac{\partial\pi_{r}^{A}}{\partial p}=a-\alpha p+\beta e(1-\xi)-(p-w)\alpha$ and $\frac{\partial^{2}\pi_{r}^{A}}{\partial p^{2}}=-2\alpha$, respectively. Because $\frac{\partial^{2}\pi_{r}^{A}}{\partial p^{2}}=-2\alpha<0$, the optimal solution must be unique. Let $\frac{\partial\pi_{r}^{A}}{\partial p}=a-\alpha p+\beta e(1-\xi)-(p-w)\alpha=0$, which yields the reaction function $\bar{p}^{A}(w,e)=\frac{\beta e(1-\xi)+w\alpha+a}{2\alpha}$. Substituting $\bar{p}^{A}(w,e)=\frac{\beta e(1-\xi)+w\alpha+a}{2\alpha}$ into $\pi_{m}^{A}=(w-c)(a-\alpha p+\beta e(1-\xi))-\frac{1}{2}ke^{2}$ yields $\pi_{m}^{A}=\frac{1}{2}(w-c)\left(a-w\alpha-\beta e(1-\xi)\right)-\frac{1}{2}ke^{2}$. Deriving the first derivatives and the second derivatives of $\pi_{m}^{A}$ with respect to $w$ and $e$ yields Hessian matrix $H_{1}=\left(\begin{matrix} -\alpha& \frac{\beta}{2}(1-\xi) \\ \frac{\beta}{2}(1-\xi) & -k \end{matrix}\right)$, where $\alpha k-\frac{1}{4}\beta^{2}(1-\xi)^{2}>0$. Then, it must be negative definite, and thus there must be a unique optimal solution. The first derivatives of $\pi_{m}^{A}$ with respect to $w$ and $e$ can are obtained as $\frac{\partial\pi_{m}^{A}}{\partial w}=\frac{a}{2}-\frac{(2w-c)\alpha}{2}+\frac{\beta e(1-\xi)}{2}$ and $\frac{\partial\pi_{m}^{A}}{\partial e}=\frac{(w-c)(1-\xi)\beta}{2}-ke$, respectively. By the first-order condition, $\frac{\partial\pi_{m}^{A}}{\partial w}=0$ and $\frac{\partial\pi_{m}^{A}}{\partial e}=0$, we obtain the optimal $e^{A*}=\frac{\beta(1-\xi)(a-\alpha c)}{4\alpha k-\beta^{2}(1-\xi)^{2}}$ and $w^{A*}=\frac{c\left(2\alpha k-\beta^{2}\left(1-\xi\right)^{2}\right)+2ak}{4\alpha k-\beta^{2}(1-\xi)^{2}}$. Then, substituting $e^{A*}$ and $w^{A*}$ into $\bar{p}^{A}(w,e)=\frac{\beta e(1-\xi)+w\alpha+a}{2\alpha}$, we can yield the optimal $p^{A*}=\frac{c\left(\alpha k-\beta^{2}\left(1-\xi\right)^{2}\right)+3ak}{4\alpha k-\beta^{2}(1-\xi)^{2}}$. Substituting the above$e^{A*}$, $w^{A*}$, and $p^{A*}$ into $\pi_{m}^{A}=(w-c)(a-\alpha p+\beta e(1-\xi))-\frac{1}{2}ke^{2}$ and $\pi_{r}^{A}=(p-w)(a+\beta e(1-\xi)-\alpha p)$, we yield the optimal $\pi_{m}^{A*}=\frac{k(a-\alpha c)^{2}}{8\alpha k-2\beta^{2}(1-\xi)^{2}}$ and $\pi_{r}^{A*}=\frac{\alpha k^{2}(a-\alpha c)^{2}}{\left(4\alpha k-(1-\xi)^{2}\beta^{2}\right)^{2}}$, respectively.

Appendix S.2: Proof of Theorem 2

Substituting $D_{B}=a-\alpha p+\beta e$ and $u=\frac{1}{2}gc_{f}^{2}$ into $\pi_{m}^{M}=(w-c-v)D_{B}+u-\frac{1}{2}ke^{2}-f$ and $\pi_{r}^{M}=(p-w)D_{B}+\frac{u}{2}$ yields $\pi_{m}^{M}=(w-c-v)(a-\alpha p+\beta e)-\frac{1}{2}ke^{2}-f+\frac{1}{2}gf^{2}$ and $\pi_{r}^{M}=(p-w)(a-\alpha p+\beta e)+\frac{1}{4}gf^{2}$. The first derivatives and the second derivatives of $\pi_{r}^{M}$ with respect to $p$ are obtained as $\frac{\partial\pi_{r}^{M}}{\partial p}=a-\alpha p+\beta e-(p-w)\alpha$ and $\frac{\partial^{2}\pi_{r}^{M}}{\partial p^{2}}=-2\alpha$, respectively. Because $\frac{\partial^{2}\pi_{r}^{M}}{\partial p^{2}}=-2\alpha<0$, there must be a unique optimal solution. Then, let $\frac{\partial\pi_{r}^{M}}{\partial p}=a-\alpha p+\beta e-(p-w)\alpha=0$, which yields the reaction function $\bar{p}^{M}(w,e)=\frac{w\alpha+\beta e+a}{2\alpha}$. Substituting $\bar{p}^{M}(w,e)=\frac{w\alpha+\beta e+a}{2\alpha}$ into $\pi_{m}^{M}=(w-c-v)(a-\alpha p+\beta e)-\frac{1}{2}ke^{2}-f+\frac{1}{2}gf^{2}$ yields $\pi_{m}^{M}=\frac{1}{2}\left(\beta e-\alpha w+a+\alpha(c+v)\right)$ $-\frac{1}{2}\left(\beta e+a\right)\left(c+v\right)-\frac{1}{2}f\left(gf-2\right)-\frac{1}{2}ke^{2}$. Deriving the first derivatives and the second derivatives of $\pi_{m}^{M}$ with respect to $w$ and $e$ yields Hessian matrix $H_{2}=\left(\begin{matrix} -\alpha& \frac{\beta}{2} \\ \frac{\beta}{2} & -k \end{matrix}\right)$, where $\alpha k-\frac{\beta^{2}}{4}>0$. Then, it must be negative definite, and thus the optimal solution must be unique. The first derivatives of $\pi_{m}^{M}$ with respect to $w$ and $e$ are obtained as $\frac{\partial\pi_{m}^{M}}{\partial w}=\frac{1}{2}\left(2\alpha w+\beta e-a+c+v\right)$ and $\frac{\partial\pi_{m}^{M}}{\partial e}=\frac{1}{2}\beta\left(w-c-v\right)-ke$, respectively. The first-order condition $\frac{\partial\pi_{m}^{M}}{\partial w}=0$ and $\frac{\partial\pi_{m}^{M}}{\partial e}=0$ yields the optimal $e^{M*}=\frac{\beta(a-\alpha(c+v))}{4\alpha k-\beta^{2}}$ and $w^{M*}=\frac{(c+v)\left(2\alpha k-\beta^{2}\right)+2ak}{4\alpha k-\beta^{2}}$. Substituting $e^{M*}$ and $w^{M*}$ into $\bar{p}^{M}(w,e)=\frac{w\alpha+\beta e+a}{2\alpha}$ yields the optimal $p^{M*}=\frac{(c+v)\left(\alpha k-\beta^{2}\right)+3ak}{4\alpha k-\beta^{2}}$. Substituting $e^{M*}$, $w^{M*}$, and $p^{M*}$ into $\pi_{r}^{M}=(p-w)(a-\alpha p+\beta e)+\frac{1}{4}gf^{2}$ and $\pi_{m}^{M}=(w-c-v)(a-\alpha p+\beta e)-\frac{1}{2}ke^{2}-f+\frac{1}{2}gf^{2}$, we can yield the optimal $\pi_{r}^{M*}=\frac{4k^{2}\alpha^{3}(c+v)^{2}+4\alpha ak^{2}\left(a-2\alpha\left(c+v\right)\right)+gf^{2}\left(8\alpha k\left(2\alpha k-\beta^{2}\right)+\beta^{4}\right)}{4\left(4\alpha k-\beta^{2}\right)^{2}}$ and $\pi_{m}^{M*}=\frac{k\left(a-(c+v)\right)^{2}+f(gf-2)(4\alpha k-\beta^{2})}{2\left(4\alpha k-\beta^{2}\right)}$, respectively.

Appendix S.3: Proof of Theorem 3

Substituting both $D_{B}=a-\alpha p+\beta e$ and $u=\frac{1}{2}gc_{f}^{2}$ into $\pi_{m}^{R}=(w-c-v)D_{B}+\frac{u}{2}-\frac{1}{2}ke^{2}$ and $\pi_{r}^{R}=(p-w)D_{B}+u-f$ yields $\pi_{m}^{R}=(w-c)\left(a-\alpha p+\beta e\right)-\frac{1}{2}ke^{2}+\frac{1}{4}gf^{2}$ and $\pi_{r}^{R}=(p-w-v)\left(a-\alpha p+\beta e\right)-f+\frac{1}{2}gf^{2}$, respectively. The first derivatives and the second derivatives of $\pi_{r}^{R}$ with respect to $p$ are obtained as $\frac{\partial\pi_{r}^{R}}{\partial p}=a-\alpha p+\beta e-(p-w-v)\alpha$ and $\frac{\partial^{2}\pi_{r}^{R}}{\partial p^{2}}=-2\alpha$, respectively. Because $\frac{\partial^{2}\pi_{r}^{R}}{\partial p^{2}}=-2\alpha<0$, the optimal solution must be unique. Let $\frac{\partial\pi_{r}^{R}}{\partial p}=a-\alpha p+\beta e-$ $(p-w-v)\alpha=0$, which leads to the reaction function  $\bar{p}^{R}(w,e)=$ $\frac{\left(v+w\right)\alpha+\beta e+a}{2\alpha}$. Substituting $\bar{p}^{R}(w,e)=\frac{\left(v+w\right)\alpha+\beta e+a}{2\alpha}$ into $\pi_{m}^{R}=(w-c)\left(a-\alpha p+\beta e\right)$ $-\frac{1}{2}ke^{2}+\frac{1}{4}gf^{2}$ yields $\pi_{m}^{R}=\frac{1}{2}(w-c)\left(a-\alpha v-w\alpha+\beta e\right)-\frac{1}{2}ke^{2}+\frac{1}{4}gf^{2}$. Deriving the first derivatives and the second derivatives of $\pi_{m}^{R}$ with respect to $w$ and $e$ yields Hessian matrix $H_{3}=\left(\begin{matrix} -\alpha& \frac{\beta}{2} \\ \frac{\beta}{2} & -k \end{matrix}\right)$, where $\alpha k-\frac{\beta^{2}}{4}>0$. Then, it must be negative definite, and thus the optimal solution must be unique. The first derivatives of $\pi_{m}^{R}$ with respect to $w$ and $e$ are obtained as $\frac{\partial\pi_{m}^{R}}{\partial w}=\frac{1}{2}\left(\beta e+a+\alpha\left(c-2w-v\right)\right)$ and $\frac{\partial\pi_{m}^{R}}{\partial e}=\frac{1}{2}(w-c)\beta-ke$, respectively. By the first-order condition $\frac{\partial\pi_{m}^{R}}{\partial w}=0$ and $\frac{\partial\pi_{m}^{R}}{\partial e}=0$, we can yield the optimal $e^{R*}=\frac{\beta\left(a-\alpha\left(c+v\right)\right)}{4\alpha k-\beta^{2}}$ and $w^{R*}=\frac{c\left(2\alpha k-\beta^{2}\right)+2k\left(a-\alpha v\right)}{4\alpha k-\beta^{2}}$, respectively. Substituting the above $e^{R*}$ and $w^{R*}$ into $\bar{p}^{R}(w,e)=\frac{\left(v+w\right)\alpha+\beta e+a}{2\alpha}$ yields $p^{R*}=\frac{\left(c+v\right)\left(\alpha k-\beta^{2}\right)+3ak}{4\alpha k-\beta^{2}}$. Furthermore, substituting the above $e^{R*}$, $w^{R*}$, and $p^{R*}$ into $\pi_{m}^{R}=(w-c)\left(a-\alpha p+\beta e\right)-\frac{1}{2}ke^{2}+\frac{1}{4}gf^{2}$ and  $\pi_{r}^{R}=(p-w-v)\left(a-\alpha p+\beta e\right)$ $-f+\frac{1}{2}gf^{2}$yields $\pi_{m}^{R*}=\frac{gf^{2}\left(4\alpha k-\beta^{2}\right)+2k\alpha^{2}\left(c+v\right)^{2}}{4\left(4\alpha k-\beta^{2}\right)}+$ $\frac{2ak\left(a-2\alpha\left(c+v\right)\right)}{4\left(4\alpha k-\beta^{2}\right)}$ and $\pi_{r}^{R*}=\frac{2k^{2}\alpha^{3}\left(c+v\right)^{2}+f(gf-2)\left(8\alpha k\left(2\alpha k-\beta^{2}\right)+\beta^{4}\right)}{2\left(4\alpha k-\beta^{2}\right)^{2}}+$ $\frac{\alpha ak^{2}\left(a-2\alpha\left(c+v\right)\right)}{\left(4\alpha k-\beta^{2}\right)^{2}}$, respectively.

Appendix S.4: Proof of Theorem 4

Substituting $D_{B}=a-\alpha p+\beta e$ and $u=\frac{1}{2}gc_{f}^{2}$ into $\pi_{m}^{MR}=(w-c-v)D_{B}+u-\frac{1}{2}ke^{2}-\frac{1}{2}f$ and $\pi_{r}^{MR}=(p-w-v)D_{B}+u-\frac{1}{2}f$ yields $\pi_{m}^{MR}=(w-c-v)\left(a-\alpha p+\beta e\right)-\frac{1}{2}ke^{2}+\frac{1}{2}f\left(gf-1\right)$ and $\pi_{r}^{MR}=(p-w-v)\left(a-\alpha p+\beta e\right)$ $+\frac{1}{2}\left(gf-1\right)$. The first derivatives and the second derivatives of $\pi_{r}^{MR}$ with respect to $p$ are obtained as $\frac{\partial\pi_{r}^{MR}}{\partial p}=\left(w+v-2p\right)\alpha+\beta e+a$ and $\frac{\partial^{2}\pi_{r}^{MR}}{\partial p^{2}}=-2\alpha$, respectively. Because $\frac{\partial^{2}\pi_{r}^{MR}}{\partial p^{2}}=-2\alpha<0$, the optimal solution must be unique. Then, let $\frac{\partial\pi_{r}^{MR}}{\partial p}=\left(w+v-2p\right)\alpha+$ $\beta e+a=0$, which leads to the reaction function  $\bar{p}^{MR}(w,e)=$ $\frac{\left(w+v\right)\alpha+\beta e+a}{2\alpha}$. Substituting $\bar{p}^{MR}(w,e)=\frac{\left(w+v\right)\alpha+\beta e+a}{2\alpha}$ into  $\pi_{m}^{MR}=\frac{1}{2}f\left(gf-1\right)-\frac{1}{2}ke^{2}$ $+(w-c-v)\left(a-\alpha p+\beta e\right)$yields  $\pi_{m}^{MR}=\frac{1}{2}\alpha\left(c-w+\frac{1}{2}v\right)\left(w+\frac{1}{2}v\right)$ $-\frac{1}{4}\left(\beta e+a\right)\left(v-2\left(w-c\right)\right)$ $-\frac{1}{2}ke^{2}$. Deriving the first derivatives and the second derivatives of $\pi_{m}^{MR}$ with respect to $w$ and $e$ yields Hessian matrix $H_{4}=\left(\begin{matrix} -\alpha& \frac{\beta}{2} \\ \frac{\beta}{2} & -k \end{matrix}\right)$, where $\alpha k-\frac{\beta^{2}}{4}>0$. Then, it must be negative definite, and thus the optimal solution must be unique. The first derivatives of $\pi_{m}^{MR}$ with respect to $w$ and $e$ are obtained as $\frac{\partial\pi_{m}^{MR}}{\partial w}=\frac{1}{2}\left(a-(2w-c)\alpha+\beta e\right)$ and $\frac{\partial\pi_{m}^{MR}}{\partial e}=\frac{1}{2}(w-c-v)\beta-ke$, respectively. By the first-order condition $\frac{\partial\pi_{m}^{MR}}{\partial w}=0$ and $\frac{\partial\pi_{m}^{MR}}{\partial e}=0$, we could attain the optimal $e^{MR*}=\frac{\beta\left(a-\alpha\left(c+2v\right)\right)}{4\alpha k-\beta^{2}}$ and $w^{MR*}=\frac{2k(\alpha c+a)-\left(c+v\right)\beta^{2}}{4\alpha k-\beta^{2}}$, respectively. Substituting $e^{MR*}$ and $w^{MR*}$ into $\bar{p}^{MR}(w,e)=\frac{\left(w+v\right)\alpha+\beta e+a}{2\alpha}$ yields $p^{MR*}=\frac{\left(c+2v\right)\left(\alpha k-\beta^{2}\right)+3ak}{4\alpha k-\beta^{2}}$. Substituting $e^{MR*}$, $w^{MR*}$, and $p^{MR*}$ into $\pi_{m}^{MR}=(w-c-b)\left(a-\alpha p+\beta e\right)-\frac{1}{2}ke^{2}+\frac{1}{2}f\left(gf-1\right)$ and $\pi_{r}^{MR}=(p-w-b)\left(a-\alpha p+\beta e\right)$ $+\frac{1}{2}\left(gf-1\right)$, yield the optimal $\pi_{m}^{MR*}=\frac{f(4\alpha k-\beta^{2})(gf-1)+k\alpha^{2}\left(c+2v\right)^{2}}{2\left(4\alpha k-\beta^{2}\right)}$ $+\frac{ak\left(a-2\alpha\left(c+2v\right)\right)}{2\left(4\alpha k-\beta^{2}\right)}$ and the optimal , respectively.

Appendix S.5: Proof of Lemma 1

From Theorems 1 and 3, we could attain $\pi_{r}^{R*}-\pi_{r}^{A*}=\frac{Q}{2\left(4\alpha k-\left(1-\xi\right)^{2}\beta^{2}\right)^{2}\left(4\alpha k-\beta^{2}\right)^{2}}$, where $Q=2k^{2}\alpha^{3}\left(4\alpha k-\beta^{2}\left(1-\xi\right)^{2}\right)^{2}v^{2}$ $+4\alpha^{2}k^{2}\left(a-\alpha c\right)\left(4\alpha k-\beta^{2}\left(1-\xi\right)^{2}\right)^{2}v$ $+C$. Here, $C$ represents a long formula which is unnecessary to be expanded because it does not influence the results at all. Let $\pi_{r}^{R*}-\pi_{r}^{A*}=0$, then $Q=0$, which can be regarded as a univariate quadratic equation in terms of $v$. Solving the equation $Q=0$, we attain its roots as following$\bar{b}_{1}(f)=\frac{Ak\alpha(a-\alpha c)-\sqrt{\alpha B^{2}\left(\alpha k^{2}(a-\alpha c)^{2}-8A^{2}f\left(gf-2\right)\right)}}{Ak\alpha^{2}}$ and $\bar{b}_{1}^{'}(f)=\frac{Ak\alpha(a-\alpha c)}{Ak\alpha^{2}}+$ $\frac{\sqrt{\alpha B^{2}\left(\alpha k^{2}(a-\alpha c)^{2}-8A^{2}f\left(gf-2\right)\right)}}{Ak\alpha^{2}}$, where $A=\alpha k-\frac{(1-\xi)^{2}\beta^{2}}{4}$ , $B=\alpha k-\frac{\beta^{2}}{4}$, and $\alpha B^{2}\left(\alpha k^{2}(a-\alpha c)^{2}-8A^{2}f\left(gf-2\right)\right)>0$. From $2k^{2}\alpha^{3}\left(4\alpha k-\beta^{2}\left(1-\xi\right)^{2}\right)^{2}>0$, the graph of the univariate quadratic function $Q(v)$ opens upward. It can be proven $\bar{b}_{1}(f)>0$ and $\bar{b}_{1}^{'}(f)>0$ by calculating. From the set precondition $v<\frac{a-2\alpha c}{4\alpha}$, we could attain $0<\bar{b}_{1}(f)<\frac{a-2\alpha c}{4\alpha}$ while $\bar{b}_{1}^{'}(f)>\frac{a-2\alpha c}{4\alpha}$, where the latter contradicts the precondition and thus should be discarded. Consequently, there must be $\pi_{r}^{R*}>\pi_{r}^{A*}$ when $v<\bar{b}_{1}(f)$.

Appendix S.6: Proof of Lemma 2

From Theorems 1 and 2, we could attain $\pi_{r}^{M*}-\pi_{r}^{A*}=\frac{P}{A\left(32\alpha k-8\beta^{2}\right)}$, where . Let $\pi_{r}^{M*}-\pi_{r}^{A*}=0$, then $P=0$, which can be regarded as a univariate quadratic equation in terms of $v$. Solving the equation $P=0$ yields its roots $\bar{b}_{2}(f)=\frac{Ak\left(a-\alpha c\right)-\sqrt{ABk\left(k\left(a-\alpha c\right)^{2}-4Ac_{f}\left(gc_{f}-2\right)\right)}}{Ak\alpha}$ and $\bar{b}_{2}^{'}(f)=\frac{Ak\left(a-\alpha c\right)+\sqrt{ABk\left(k\left(a-\alpha c\right)^{2}-4Ac_{f}\left(gc_{f}-2\right)\right)}}{Ak\alpha}$, where $A=\alpha k-\frac{(1-\xi)^{2}\beta^{2}}{4}$ , $B=\alpha k-\frac{\beta^{2}}{4}$, and $ABk\left(k\left(a-\alpha c\right)^{2}-4Af\left(gf-2\right)\right)>0$. From $k\alpha^{2}\left(4\alpha k-\left(1-\xi\right)^{2}\beta^{2}\right)>0$, the graph of the univariate quadratic function $P(v)$ opens upward. It can be proven $\bar{b}_{2}(f)>0$ and $\bar{b}_{2}^{'}(f)>0$ by calculating. From the set precondition $v<\frac{a-2\alpha c}{4\alpha}$, we could attain $0<\bar{b}_{2}(f)<\frac{a-2\alpha c}{4\alpha}$ while $\bar{b}_{2}^{'}(f)>\frac{a-2\alpha c}{4\alpha}$, where the latter contradicts the precondition and should be discarded. Consequently, there must be $\pi_{r}^{M*}>\pi_{r}^{A*}$ when $v<\bar{b}_{2}(f)$.

Appendix S.7: Proof of Lemma 3

From Theorems 2 and 4, we could attain $\pi_{r}^{MR*}-\pi_{r}^{M*}=\frac{S}{4\left(4\alpha k-\beta^{2}\right)^{2}}$, where . Let $\pi_{r}^{MR*}$ $-\pi_{r}^{M*}=0$, then $S=0$, which can be regarded as a univariate quadratic equation in terms of $v$. Solving the above equation$S=0$, we can yield its roots $\bar{b}_{3}(f)=\frac{\alpha k\left(a-\alpha c\right)-\sqrt{\alpha\left(\alpha k^{2}(a-\alpha c)^{2}-12fB^{2}\left(gf-2\right)\right)}}{3k\alpha^{2}}$ and $\bar{b}_{3}^{'}(f)=\frac{\alpha k\left(a-\alpha c\right)+\sqrt{\alpha\left(\alpha k^{2}(a-\alpha c)^{2}-12fB^{2}\left(gf-2\right)\right)}}{3k\alpha^{2}}$, where $A=\alpha k-\frac{(1-\xi)^{2}\beta^{2}}{4}$, $B=\alpha k-\frac{\beta^{2}}{4}$, and $\alpha\left(\alpha k^{2}(a-\alpha c)^{2}-12fB^{2}\left(gf-2\right)\right)>0$. From $12k^{2}\alpha^{3}>0$, the graph of the univariate quadratic function $S(v)$ opens upward. It can be proven $\bar{b}_{3}(f)>0$ and $\bar{b}_{3}^{'}(f)>0$ by calculating. From the set precondition $v<\frac{a-2\alpha c}{4\alpha}$, we could attain $0<\bar{b}_{3}(f)<\frac{a-2\alpha c}{4\alpha}$ while $\bar{b}_{3}^{'}(f)>\frac{a-2\alpha c}{4\alpha}$, where the latter contradicts the precondition and should be discarded. Consequently, there must be $\pi_{r}^{MR*}>\pi_{r}^{M*}$ when $v<\bar{b}_{3}(f)$.

Appendix S.8: Proof of Lemma 4

From Theorems 3 and 4, we could attain $\pi_{m}^{MR*}-\pi_{m}^{R*}=\frac{R}{16\alpha k-4\beta^{2}}$, where . Let $\pi_{r}^{MR*}-\pi_{r}^{R*}=0$, then $R=0$, which can be regarded as a univariate quadratic equation in terms of $v$. Solving the equation $R=0$ yields its roots $\bar{b}_{4}(f)=\frac{k(a-\alpha c)-\sqrt{k\left(k(a-\alpha c)^{2}-6Bf\left(gf-2\right)\right)}}{3k\alpha}$ and $\bar{b}_{4}^{'}(f)=\frac{k(a-\alpha c)+\sqrt{k\left(k(a-\alpha c)^{2}-6Bf\left(gf-2\right)\right)}}{3k\alpha}$, where $A=\alpha k-\frac{(1-\xi)^{2}\beta^{2}}{4}$ , $B=\alpha k-\frac{\beta^{2}}{4}$, and $k\left(k(a-\alpha c)^{2}-6Bf\left(gf-2\right)\right)>0$. From $6\alpha^{2}k>0$, the graph of the univariate quadratic function $R(v)$ opens upward. It can be proven $\bar{b}_{4}(f)>0$ and $\bar{b}_{4}^{'}(f)>0$ by calculating. From the set precondition $v<\frac{a-2\alpha c}{4\alpha}$, we could attain $0<\bar{b}_{4}(f)<\frac{a-2\alpha c}{4\alpha}$ while $\bar{b}_{4}^{'}(f)>\frac{a-2\alpha c}{4\alpha}$, where the latter contradicts the precondition and should be discarded. Consequently, there must be $\pi_{r}^{MR*}>\pi_{r}^{R*}$ when $v<\bar{b}_{4}(f)$.

Appendix S.9: Proof of Lemma 5

First, we could attain$\bar{b}_{1}(f)-\bar{b}_{2}(f)=\frac{1}{Ak\alpha^{2}}\left(\sqrt{ABk\alpha^{2}\left(k(a-\alpha c)^{2}-4Af(gf-2)\right)}\right.-$ $\left.\sqrt{\alpha B^{2}-8A^{2}f\left(gf-2\right)+\alpha k^{2}\left(a-\alpha c\right)^{2}}\right)$, where $\frac{1}{Ak\alpha^{2}}>0$. Then, $sign\left(\bar{b}_{1}(f)-\bar{b}_{2}(f)\right)=$ $sign\left(ABk\alpha^{2}\left(k(a-\alpha c)^{2}-4Af(gf-2)\right)-\left(\alpha B^{2}-8A^{2}f\left(gf-2\right)+\alpha k^{2}\left(a-\alpha c\right)^{2}\right)\right)=sign(K)$. By calculating, yield $K=B\alpha^{2}k^{2}(a-\alpha c)^{2}\left(\frac{1}{2}\beta^{2}\left(1-\left(1-\xi\right)^{2}\right)\right)+4\alpha fBA^{2}\left(gf-2\right)\left(\alpha k-\frac{1}{2}\beta^{2}\right)$, where $B\alpha^{2}k^{2}(a-\alpha c)^{2}\left(\frac{1}{2}\beta^{2}\left(1-\left(1-\xi\right)^{2}\right)\right)>0$ and $4\alpha fBA^{2}\left(gf-2\right)\left(\alpha k-\frac{1}{2}\beta^{2}\right)>0$. Then, $sign\left(\bar{b}_{1}(f)-\bar{b}_{2}(f)\right)=1$, namely, $\bar{b}_{1}(f)>\bar{b}_{2}(f)$.

Second, from $\frac{\partial(\bar{b}_{2}(f)-\bar{b}_{3}(f))}{\partial\xi}=\frac{k\beta^{2}B(1-\xi)(a-\alpha c)^{2}}{4A\alpha\sqrt{ABk\left(k(a-\alpha c)^{2}-4Af\left(gf-2\right)\right)}}>0$, we could attain$\bar{b}_{2}(f)-\bar{b}_{3}(f)$ minimizes at $\xi=0$, by which $\left.\left(\bar{b}_{2}(f)-\bar{b}_{3}(f)\right)\right|_{\min}=\left.\left(\bar{b}_{2}(f)-\bar{b}_{3}(f)\right)\right|_{\xi=0}=$ $\frac{1}{3k\alpha^{2}}\left(\sqrt{\alpha^{2}k^{2}\left(a-\alpha c\right)^{2}-12\alpha fB^{2}(gf-2)}\right.$ $\left.+2\alpha k\left(a-\alpha c\right)-3\sqrt{\alpha^{2}k^{2}\left(a-\alpha c\right)^{2}-4\alpha^{2}kBf(gf-2)}\right)$, where $\frac{1}{3Bk\alpha^{2}}>0$. Furthermore, from$3\alpha fB^{2}(gf-2)<\alpha^{2}kBf(gf-2)$, we also could attain $\sqrt{\alpha^{2}k^{2}\left(a-\alpha c\right)^{2}-12\alpha fB^{2}(gc_{f}-2)}>\sqrt{\alpha^{2}k^{2}\left(a-\alpha c\right)^{2}-4\alpha^{2}kBf(gf-2)}$, based on which we can yield$\sqrt{\alpha^{2}k^{2}\left(a-\alpha c\right)^{2}-12B\alpha f(gf-2)}-3\sqrt{\alpha^{2}k^{2}\left(a-\alpha c\right)^{2}-4\alpha^{2}kBf(gf-2)}+2\alpha k\left(a-\alpha c\right)>$ $-2\sqrt{\alpha^{2}k^{2}\left(a-\alpha c\right)^{2}-4\alpha^{2}kBf(gf-2)}+2\alpha k\left(a-\alpha c\right)>0$. Then, $\left.\left(\bar{b}_{2}(f)-\bar{b}_{3}(f)\right)\right|_{\min}>0$, by which $\bar{b}_{2}(f)-\bar{b}_{3}(f)>0$. Consequently, $\bar{b}_{2}(f)>\bar{b}_{3}(f)$.

Third, by calculating, we could attain $\bar{b}_{3}(f)-\bar{b}_{4}(f)=$ $\frac{1}{3k\alpha^{2}}\left(\alpha\sqrt{k\left(k(a-\alpha c)^{2}-6Bf\left(gf-2\right)\right)}\right.$ $\left.-\sqrt{\alpha\left(\alpha k^{2}(a-\alpha c)^{2}-12fB^{2}\left(gf-2\right)\right)}\right)$, where $\frac{1}{3k\alpha^{2}}>0$. Moreover, it can be proven $\alpha\sqrt{k\left(k(a-\alpha c)^{2}-6Bf\left(gf-2\right)\right)}>$ $\sqrt{\alpha\left(\alpha k^{2}(a-\alpha c)^{2}-12fB^{2}\left(gf-2\right)\right)}$. Then, $\bar{b}_{3}(f)-\bar{b}_{4}(f)>0$, namely, $\bar{b}_{3}(f)>\bar{b}_{4}(f)$.

Summarizing the above three aspects, $\bar{b}_{1}(f)>\bar{b}_{2}(f)>\bar{b}_{3}(f)>\bar{b}_{4}(f)$.
